# Graph of graphs analysis for multiplexed data with application to imaging mass cytometry

**DOI:** 10.1371/journal.pcbi.1008741

**Published:** 2021-03-29

**Authors:** Ya-Wei Eileen Lin, Tal Shnitzer, Ronen Talmon, Franz Villarroel-Espindola, Shruti Desai, Kurt Schalper, Yuval Kluger

**Affiliations:** 1 Viterbi Faculty of Electrical Engineering, Technion - Israel Institute of Technology, Haifa, Israel; 2 Department of Pathology, School of Medicine, Yale University, New Haven, Connecticut, United States of America; 3 Department of Medicine, Yale School of Medicine and Yale Cancer Center, New Haven, Connecticut, United States of America; 4 Computational Biology and Bioinformatics Program, Yale University, New Haven, Connecticut, United States of America; 5 Program of Applied Mathematics, Yale University, New Haven, Connecticut, United States of America; Carnegie Mellon University, UNITED STATES

## Abstract

Imaging Mass Cytometry (IMC) combines laser ablation and mass spectrometry to quantitate metal-conjugated primary antibodies incubated in intact tumor tissue slides. This strategy allows spatially-resolved multiplexing of dozens of simultaneous protein targets with 1*μm* resolution. Each slide is a spatial assay consisting of high-dimensional multivariate observations (*m*-dimensional feature space) collected at different spatial positions and capturing data from a single biological sample or even representative spots from multiple samples when using tissue microarrays. Often, each of these spatial assays could be characterized by several regions of interest (ROIs). To extract meaningful information from the multi-dimensional observations recorded at different ROIs across different assays, we propose to analyze such datasets using a two-step graph-based approach. We first construct for each ROI a graph representing the interactions between the *m* covariates and compute an *m* dimensional vector characterizing the steady state distribution among features. We then use all these *m*-dimensional vectors to construct a graph between the ROIs from all assays. This second graph is subjected to a nonlinear dimension reduction analysis, retrieving the intrinsic geometric representation of the ROIs. Such a representation provides the foundation for efficient and accurate organization of the different ROIs that correlates with their phenotypes. Theoretically, we show that when the ROIs have a particular bi-modal distribution, the new representation gives rise to a better distinction between the two modalities compared to the maximum a posteriori (MAP) estimator. We applied our method to predict the sensitivity to PD-1 axis blockers treatment of lung cancer subjects based on IMC data, achieving 97.3% average accuracy on two IMC datasets. This serves as empirical evidence that the graph of graphs approach enables us to integrate multiple ROIs and the intra-relationships between the features at each ROI, giving rise to an informative representation that is strongly associated with the phenotypic state of the entire image.

## Author summary

We propose a two-step graph-based analyses for high-dimensional multiplexed datasets characterizing ROIs and their inter-relationships. The first step consists of extracting the steady state distribution of the random walk on the graph, which captures the mutual relations between the covariates of each ROI. The second step employs a nonlinear dimensionality reduction on the steady state distributions to construct a map that unravels the intrinsic geometric structure of the ROIs. We theoretically show that when the ROIs have a two-class structure, our method accentuates the distinction between the classes. Particularly, in a setting with Gaussian distribution it outperforms the MAP estimator, implying that the mutual relations between the covariates within the ROIs and spatial coordinates are well captured by the steady state distributions. We apply our method to imaging mass cytometry (IMC). Our analysis provides a representation that facilitates prediction of the sensitivity to PD-1 axis blockers treatment of lung cancer subjects. Particularly, our approach achieves state of the art results with average accuracy of 97.3% on two IMC datasets.

This is a *PLOS Computational Biology* Methods paper.

## Introduction

Consider multi-feature observations collected at different spatial positions. Data structure of this type requires analysts to address two immediate natural questions. First is how to characterize the associations between the different features in each position. Second is how to organize the observations from different spatial positions into an informative representation.

We approach these two questions from the standpoint of manifold learning, which is a class of nonlinear dimensionality reduction techniques for high-dimensional data [1–4]. The common assumption in manifold learning is that the multi-feature observations lie on a hidden lower-dimensional manifold. Such an assumption facilitates the incorporation of geometric concepts such as metrics, geodesic distances, and embedding, into useful data analysis techniques. In order to learn a (continuous) manifold from discrete data samples, commonly-used manifold learning methods rely on the construction of a graph. Typically, the data samples form the graph nodes and the edges of the graph are determined according to some similarity notion that is usually application-specific.

In our work, we adhere to manifold learning techniques and propose a method consisting of two-step graph analysis. At the first stage, we build a graph for each spatial position, where the graph nodes are the multi-feature observations. The motivation to build such a graph rather than using the observations directly stems from an assumption that the information about the sample at each spatial position is better expressed by the mutual-relations between the features. Then, we define a random walk on this graph and build a characteristic vector of the respective spatial position by computing the steady state distribution (SSD) of the random walk. For analysis purposes, we define a new notion of heterogeneity, representing a statistical diversity of the multiple features, and show that the SSD characterizes each spatial position in terms of this heterogeneity. In addition, using this notion of heterogeneity, when the density of the observations at the spatial positions is bi-modal, we show that these SSDs can lead to an accurate identification of the two modes, outperforming the maximum a-posteriori (MAP) estimator [5] in a statistical setting with Gaussian distributions.

At the second stage, we build a graph whose nodes are the new characteristic vectors (SSDs) of all the spatial positions. We apply diffusion maps [4] to this second graph and obtain a low dimensional representation of the spatial positions. The dimension of the computed representation is determined by a nonlinear variant of the Jackstraw algorithm [6].

Broadly, the proposed algorithm could be viewed as building a *graph of graphs*. From a manifold learning standpoint, this two-step procedure could be viewed as inferring a *manifold of manifolds*. Namely, at the first stage, we recover the local manifolds that underlie the multiple features at each spatial location, and then, at the second stage, we recover the global manifold between the spatial positions, formed by the collection of all local manifolds. This standpoint is related to a large body of recent work involving the discovery and analysis of multi-manifold structures, e.g., alternating diffusion [7–10], multi-view diffusion maps [11], joint Laplacian diagonalization [12], to name just a few. Therefore, the proposed method can be viewed as a follow up work along this line of research.

We apply our method to imaging mass cytometry (IMC) [13–15]. IMC is a new technique for multiplexed simultaneous imaging of proteins and protein modifications at subcellular resolution, ideally suited to uncover molecular and structural alterations of diseased tissues such as in cancer. IMC analysis can also be used to study the composition of non-diseases tissue samples such as histology studies or molecular profiles. The acquired intensities of the protein expression levels are viewed as markers, providing important biological information on the tissues of interest. This acquisition procedure gives rise to multi-feature observations at different spatial positions, where the multiple features are the markers and a selected subset of the spatial positions are ROIs within pathology slides.

Our experimental study focuses on one of the important tasks of IMC data analysis: associating the response status of a patient to a therapeutic intervention with a high-dimensional spatial IMC sample from the relevant patients’ tissues. Here, we propose to recast this problem as a binary hypothesis testing problem. We assume that all the ROIs of each patient can be labeled by the patient’s response or non-response status. The collection of all ROIs from the patients’ cohort induces a bi-modal density of expression signatures. Then, given the protein expression levels within the ROIs of a certain tissue type, we ask whether the subject was responsive to treatment. We showcase the performance of the proposed method on two IMC cohorts consisting of samples taken from lung cancer subjects. We achieve a average 97.3% prediction accuracy of response to treatment (PD-1 axis blockers) in an unsupervised manner. This result outperforms competing methods, specifically, the results obtained by (i) diffusion maps (DM) [4] directly applied to the multi-feature observations, (ii) the heat kernel signature (HKS) [16], and (iii) the wave kernel signature (WKS) [17].

## Results

### Ethics statement

The study was approved by the Yale University Human Investigation Committee protocols #9505008219 and #1608018220; or local institutional protocols which approved the patient consent forms or, in some cases waiver of consent when retrospectively collected archive tissue was used in a de-identified manner.

### Overview

We start by presenting the problem setting. Consider *n* data points ${\left\{x_{i}\right\}}_{i=1}^{n}$ from a hidden manifold M embedded in a high-dimensional Euclidean space $R^{k}$. Assume we do not have direct access to these data points; instead, these data points are measured through *m* observation functions $f_{j}:M\to R^{d}$, where *j* = 1, …, *m* is the index of the observation function. Given *n* multi-feature observations *f*_*j*_(*x*_*i*_) of the data point *x*_*i*_ for *i* = 1, …, *n*, each consisting of *m* features *j* = 1, …, *m*, our goal is to recover the data points *x*_*i*_ on the hidden manifold M.

In the context of IMC, the data points represent the treatment outcome based on *n* spatial positions located at ROIs within pathology slides of tissues from several patients. At each spatial position *i* = 1, …, *n*, the observations *f*_*j*_(*x*_*i*_) for *j* = 1, …, *m* are the expression levels of *m* markers. Each observation $f_{j}\left(x_{i}\right)\in R^{d}$ is a patch of *d* pixels of the expression level image of marker *j* at position *i*.

To simplify the presentation of our approach, we begin with an illustrative localization problem, which is simpler than the IMC problem. Suppose we have a surface M and objects located at *x*_*i*_ on M. The locations *x*_*i*_ are hidden, but measured through *m* sensors, such that for each location *x*_*i*_ we have *m* multi-feature observations ${\left\{f_{j}\left(x_{i}\right)\right\}}_{j=1}^{m}$. That is *f*_*j*_(*x*_*i*_) is a *d*-dimensional observation of sensor *j* when the object is at *x*_*i*_. The goal is to recover the locations *x*_*i*_ on the surface M given ${\left\{f_{j}\left(x_{i}\right)\right\}}_{j=1}^{m}$.

Our approach consists of two stages. At the first stage, we construct a graph for each data point *x*_*i*_ in order to capture associations between its *m* multi-feature observations ${\left\{f_{j}\left(x_{i}\right)\right\}}_{j=1}^{m}$. Capturing such mutual-relationships is natural in the context of localization problems, e.g., the triangulation property [18] in which the relative locations of the sensors are exploited. In addition, these mutual-relations are typically more robust to noise in comparison with the nominal values of the multi-feature observations, *f*_*j*_(*x*_*i*_), themselves. Concretely, consider the *m* observations ${\left\{f_{j}\left(x_{i}\right)\right\}}_{j=1}^{m}$ associated with the data point *x*_*i*_. Each observation *f*_*j*_(*x*_*i*_) forms a single node in the graph, hereby denoted as node *j*, giving rise to a graph with a total of *m* nodes. The graph we consider is the complete graph, where the weights of the edges are determined based on the Euclidean distance between the corresponding observations: the weight of the edge connecting nodes *j* and *k* is proportional to exp{−‖*f*_*j*_(*x*_*i*_) − *f*_*k*_(*x*_*i*_)‖^2^}. Then, we compute the SSD of a random walk defined on this graph at each location. SSD has a vector form that embodies the multi-feature inter-relationships of the data point *x*_*i*_.

At the next step, we define a second graph based on the SSDs, characterizing the points ${\left\{x_{i}\right\}}_{i=1}^{n}$. Concretely, each data point *x*_*i*_ is represented by a node, and the pairwise distances between the SSDs form the adjacency matrix of the graph. Then, we apply a particular manifold learning technique, diffusion maps [4], to this graph. This application facilitates the recovery of the hidden manifold M in the sense that an embedding of the points *x*_*i*_ is learned, such that the distances between the embedded points respect a notion of an intrinsic distance (the diffusion distance [4]) on M. The application of diffusion maps to the second graph in the context of the localization problem gives rise to an embedding that serves as an accurate representation of the hidden locations of the data point. In Localization toy problem, we demonstrate the proposed method on several simulations of localization toy problems.

We remark that the IMC problem and the localization problem share many aspects. For example, in both problems, the multi-feature observations are noisy and the mutual-relationship between them carry important information. Yet, there is a particular aspect that makes the IMC problem more challenging; while the points on the hidden manifold in a localization problem are homogeneous because they all represent location coordinates, the points in the IMC problem could be significantly different due to the large variability in the tissue structure. Importantly, the proposed method accommodates the joint processing of such different points through their representation by the SSD.

### Proposed method

The first step of the proposed method is to construct an undirected weighted graph $G_{i}=\left(V_{i},E_{i},W_{i}\right)$ for each data point $x_{i}\in M\subset R^{k}$, where the vertex set is $V_{i}=\left\{f_{1}\left(x_{i}\right),f_{2}\left(x_{i}\right),...,f_{m}\left(x_{i}\right)\right\}$, the edge set is $E_{i}\subseteq V_{i}\times V_{i}$, and the graph weights matrix $W_{i}\in R^{m\times m}$ is given by the Gaussian kernel

$$\begin{array}{c} W_{i}\left(k,l\right)=\exp(-\frac{\left|\right|f_{k}\left(x_{i}\right)-f_{l}\left(x_{i}\right){\left|\right|}_{2}^{2}}{2\epsilon }), \end{array}$$ (1)

where *k*, *l* ∈ {1, …, *m*} and *ϵ* > 0 is a scale parameter. Note that since ***W***_*i*_ is symmetric, ***W***_*i*_ is diagonalizable. That is, there is a set of real eigenvalues ${\left\{\lambda_{j}\right\}}_{j=1}^{m}$ with a corresponding orthonormal basis of eigenvectors ${\left\{v_{j}\right\}}_{j=1}^{m}$ such that

$$\begin{array}{c} W_{i}\left(k,l\right)=\sum_{j=1}^{m}\lambda_{j}v_{j}\left(k\right)v_{j}\left(l\right). \end{array}$$ (2)

Next, we define a random walk on the graph $G_{i}$. Let $P_{i}\in R^{m\times m}$ be a row stochastic matrix given by

$$\begin{array}{c} P_{i}=D_{i}^{-1}W_{i}, \end{array}$$ (3)

where ***D***_*i*_ is a diagonal matrix whose diagonal elements are given by $D_{i}\left(k,k\right)=\sum_{l=1}^{m}W_{i}\left(k,l\right)$. The value of *P*_*i*_(*k*, *l*) can be interpreted as a transition probability from a vertex *f*_*k*_(*x*_*i*_) to a vertex *f*_*l*_(*x*_*i*_) in one step of a random walk on the graph $G_{i}$.

The transition probability matrix ***P***_*i*_ is self-adjoint and compact, and therefore, the spectral decomposition of $P_{i}^{t}$ for *t* > 0 is given by

$$\begin{array}{c} P_{i}^{t}\left(k,l\right)=\sum_{j=1}^{m}\lambda_{j}^{t}\psi_{j}\left(k\right)\phi_{j}\left(l\right), \end{array}$$ (4)

where ${\left\{\psi_{j},\phi_{j}\right\}}_{j=1}^{m}$ are the right- and left-eigenvectors with the corresponding eigenvalues ${\left\{\lambda_{j}\right\}}_{j=1}^{m}$. By the construction of ***P***_*i*_ from ***W***_*i*_, the relations between their respective eigenvectors are given by

$$\begin{array}{c} \psi_{j}\left(k\right)=\frac{v_{j}\left(k\right)}{\sqrt{D_{i}\left(k,k\right)}} \end{array}$$ (5)

and

$$\begin{array}{c} \phi_{j}\left(k\right)=v_{j}\left(k\right)\sqrt{D_{i}\left(k,k\right)}. \end{array}$$ (6)

Interestingly, in the special case where *t* = 1, the probability of the node *f*_*k*_(*x*_*i*_) to stay in place is given by

$$\begin{array}{c} P_{i}\left(k,k\right)=\sum_{j=1}^{m}\lambda_{j}v_{j}^{2}\left(k\right)=\frac{1}{D_{i}\left(k,k\right)}. \end{array}$$ (7)

Note that $\phi_{1}\in R^{m}$ is the left eigenvector of ***P***_*i*_ corresponding to λ_1_ = 1, satisfying:

$$\begin{array}{c} P_{i}^{⊤}\phi_{1}=\phi_{1}, \end{array}$$ (8)

where $P_{i}^{⊤}$ is the transpose of the matrix ***P***_*i*_. Consider an arbitrary distribution vector $\pi_{0}\in R^{m}$ and observe the expansion:

$$\begin{array}{c} \pi_{0}^{⊤}P_{i}^{t}=\sum_{j=1}^{m}\lambda_{j}^{t}\left\langle \pi_{0},\psi_{j}\right\rangle \phi_{j}^{⊤}, \end{array}$$ (9)

where $\langle \pi_{0},\psi_{j}\rangle =\pi_{0}^{⊤}\psi_{j}$ is the standard Euclidean product. In the limit *t* → ∞, $\lambda_{j}^{t}\to 0$ for *j* > 1 and therefore

$$\begin{array}{c} \pi_{0}^{⊤}P_{i}^{t}⟶\lambda_{1}^{t}\left\langle \pi_{0},\psi_{1}\right\rangle \phi_{1}^{⊤}=\phi_{1}^{⊤}≜\pi_{i}^{⊤}, \end{array}$$ (10)

where $\pi_{i}\in R^{m}$ since λ_1_ = 1, ***ψ***_1_ = **1**, and $\sum_{j=1}^{m}\pi_{0}\left(j\right)=1$. Since the random walk defined by ***P***_*i*_ is irreducible, finite, and aperiodic [19], the stationary distribution ***π***_*i*_ is a *unique* stationary distribution. The convergence in Eq (10) and the uniqueness allow us to treat the stationary distribution in this case as the steady state distribution (SSD). Note that the SSD ***π***_*i*_ ∝ ***D***_*i*_**1**, where $1\in R^{m}$ is an all-ones vector. In other words, ***π***_*i*_ can be viewed as a normalized degrees vector of the graph $G_{i}$.

We will use ***π***_*i*_ as a new characteristic vector, or a signature, of *x*_*i*_, and consequently, the induced pairwise distances ||***π***_*i*_ − ***π***_*i*′_||, where *i*, *i*′ ∈ 1, …, *n*, will be used as the desired distances between the respective graphs $G_{i}$ and $G_{i^{\prime }}$ for recovering M. At first glance, using ***π***_*i*_ may seem too simplistic. Instead, one could use the broad spectral information. Consequently, define the Diffusion Kernel Signature (DKS) by

$$\begin{array}{c} x_{i}\mapsto [{\text{DKS}}_{t}(f_{1}\left(x_{i}\right)),{\text{DKS}}_{t}(f_{2}\left(x_{i}\right)),\ldots ,{\text{DKS}}_{t}(f_{m}\left(x_{i}\right))], \end{array}$$ (11)

where

$$\begin{array}{c} {\text{DKS}}_{t}(f_{k}\left(x_{i}\right))=\sum_{j=1}^{m}\lambda_{j}^{t}\psi_{j}^{2}\left(k\right). \end{array}$$ (12)

Since λ_*j*_ is in descending order and in [0, 1], the weight they assign to the eigenvectors in (12) becomes smaller as *t* increases. As a result, the DKS can be viewed as a low-pass filter, which controls the spectral bandwidth. In addition, the DKS can be recast in terms of the diffusion distance, a notion of distance induced by diffusion maps [4] that was shown useful in a broad range of applications, e.g., [20–22]. For more details see Diffusion maps. Specifically, when *t* = 1, we can show that

$$\begin{array}{c} \begin{array}{cc} {\text{DKS}}_{t=1}(f_{k}\left(x_{i}\right)) & =\sum_{j=1}^{m}\lambda_{j}\psi_{j}^{2}\left(k\right)\\ & =\sum_{j=1}^{m}\lambda_{j}{\left(\frac{v_{j}\left(k\right)}{\sqrt{D_{i}\left(k,k\right)}}\right)}^{2}\\ & =\frac{1}{D_{i}\left(k,k\right)}\sum_{j=1}^{m}\lambda_{j}v_{j}^{2}\left(k\right)\\ & =\frac{1}{\pi_{i}^{2}\left(k\right)}, \end{array} \end{array}$$ (13)

indicating that the SSD is a special case of DKS.

We note that DKS has already appeared in previous work in the context of spectral distances in [23, 24], where it was shown that it describes the underlying geometry of M. We show in the following that the seemingly simple SSD, despite the lack of broad spectral information as in the DKS, still carries substantial information.

Note that *ϵ* is a scale parameter of the Gaussian kernel, where it can be used to infer locality. If *ϵ* is set to a small value, then ***π***_*i*_ captures local properties. Conversely, if *ϵ* is large, then ***π***_*i*_ represents the global structure. As a result, a multiscale signature can be formed, consisting of multiple SSDs *π*_*i*_ computed with different values of *ϵ*.

The final stage of our method is building a low-dimensional representation of all the data points ${\left\{x_{i}\right\}}_{i=1}^{n}$. To this end, we apply diffusion maps to the corresponding characteristic vectors (signatures) ${\left\{\pi_{i}\right\}}_{i=1}^{n}$ as follows. First, we build a global graph **G**^(2)^ whose nodes are ***π***_*i*_ and edge weights are determined by a Gaussian kernel based on the *l*_1_ *distance* between ***π***_*i*_.

That is, the global graph weights matrix **W**^(2)^ is defined by

$$\begin{array}{c} W^{\left(2\right)}\left(k,l\right)=\exp(-\frac{\left|\right|\pi_{k}-\pi_{l}{\left|\right|}_{1}^{2}}{2\epsilon^{\prime }}), \end{array}$$ (14)

where *k*, *l* ∈ [1, *n*], ||⋅||_1_ is the *l*_1_ distance and *ϵ*′ > 0 is a scale parameter. We remark that common practice is to use the *l*_2_ distance in the Gaussian kernel. The reason we use the *l*_1_ distance is described in Binary hypothesis testing, which indeed leads to better empirical performance reported in Imaging mass cytometry (IMC).

Second, we construct a random walk, denoting its transition probability matrix by ***P***^(2)^. Third, we apply the eigendecomposition to ***P***^(2)^. Fourth, we set the dimension of the new representation according to the variant of the Jackstraw method [6], as shown in Determining the dimension of data.

**Fig 1:**
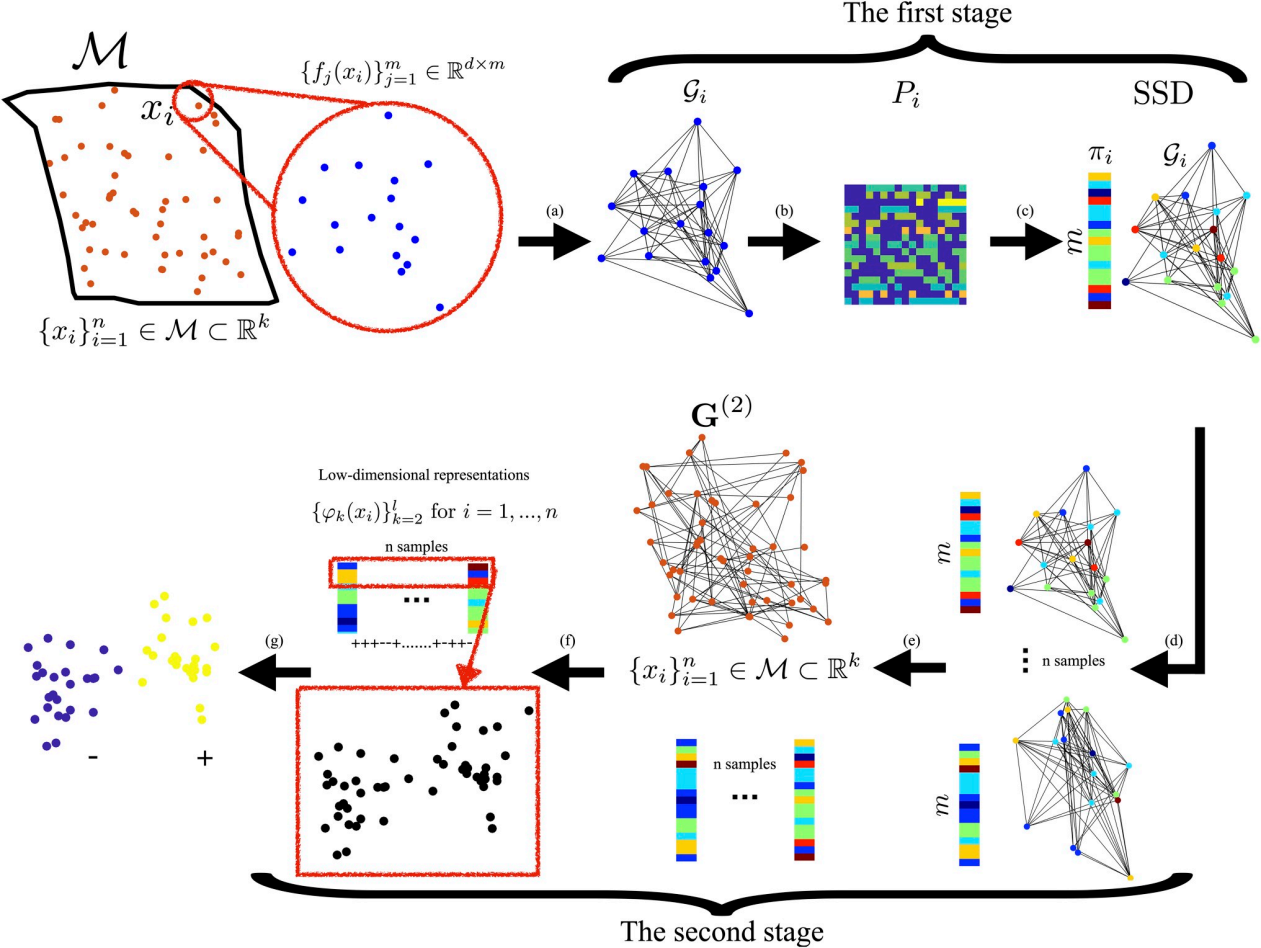
Illustrative diagram of the proposed method. (a) For each data point *x*_*i*_, we build a local graph $G_{i}$ based on its multi-feature observations ${\left\{f_{j}\left(x_{i}\right)\right\}}_{j=1}^{m}$. (b) We construct a random walk with transition probabilities matrix ***P***_*i*_ on $G_{i}$. (c) We extract the SSD signature ***π***_*i*_ from ***P***_*i*_. (e) We collect the SSDs of ${\left\{x_{i}\right\}}_{i=1}^{n}$ into an SSD representation matrix. (g) Subsequently, the matrix is subjected to a nonlinear dimensionality reduction using diffusion maps by the construction of the global graph **G**^(2)^ and the corresponding random walk with **P**^(2)^. (f) Via eigenvalue decomposition, we obtain a low-dimensional representation **Ψ**(*x*_*i*_) for *i* = 1, …, *n*, which is used in the subsequent tasks (g).

The entire method is summarized in Box 1 and a block diagram is illustrated in Fig 1.

**Box 1. A summary of the proposed method**

**Input**: A set of multi-feature observations {*f*_*j*_(*x*_*i*_)} for *i* = 1, …, *n* and *j* = 1, …, *m*.

**Output**: *l*-dimensional representation $\Psi \left(x_{i}\right)\in R^{l}$ for *i* = 1, …, *n*.

1. For each *x*_*i*_:
  - Construct a local graph $G_{i}$ with vertex set
    $$V_{i}=\left\{f_{1}\left(x_{i}\right),f_{2}\left(x_{i}\right),...,f_{m}\left(x_{i}\right)\right\},$$
    edge set $E_{i}\subseteq V_{i}\times V_{i}$, and edge weights matrix $W_{i}\in R^{m\times m}$ given in Eq (1).
  - Build a random walk on the local graph $G_{i}$ with transition probability matrix **P**_*i*_ defined in (3).
  - Compute the SSD $\pi_{i}\in R^{m}$ of ***P***_*i*_.
2. Construct a global graph ***G***^(2)^ with vertex set ${\left\{\pi_{i}\right\}}_{i=1}^{n}\in R^{m\times n}$ and the graph weights matrix **P**^(2)^ given in Eq (14).
3. Build a random walk on **G**^(2)^ with transition probability matrix **P**^(2)^.
4. Apply eigenvalue decomposition to **P**^(2)^ and obtain the eigenvectors ${\left\{\varphi_{k}\right\}}_{k=1}^{n}$.
5. Determine the number of dimensions *l* as described in Determining the dimension of data.
6. Build the mapping: $x_{i}\mapsto {\left(\varphi_{1}\left(x_{i}\right),\varphi_{2}\left(x_{i}\right),\ldots ,\varphi_{l}\left(x_{i}\right)\right)}^{⊤}≜\Psi \left(x_{i}\right)$ for *i* = 1, …, *n*.

### Theoretical analysis

We propose a statistical model that allows for a tractable analysis, showing the advantages of the SSD signature. Consider a data point $x_{i}\in M$ and denote the set of the observations by $S={\left\{f_{j}\left(x_{i}\right)\right\}}_{j=1}^{m}$. Assume the *j*-th observation $f_{j}\left(x_{i}\right)\in R^{d}$ is a realization of a *d*-dimensional random vector $V_{j}^{i}$ following a multivariate normal distribution given by

$$\begin{array}{c} V_{j}^{i}∼N\left(\mu_{j}^{i}1_{d},\sigma_{j}^{i}I_{d}\right). \end{array}$$ (15)

By collecting the *m* observations ${\left\{f_{j}\left(x_{i}\right)\right\}}_{j=1}^{m}$ and denoting the covariance matrix between the *k* and *l* observation functions by $\Sigma_{k,l}^{i}$, we obtain an *md*-dimensional vector that can be viewed as a realization of the random vector $V^{i}=\left(V_{1}^{i},\ldots ,V_{m}^{i}\right)$ with the corresponding multivariate Gaussian distribution given by

$$\begin{array}{c} N(\mu^{i},\Sigma^{i}), \end{array}$$ (16)

where

$$\mu^{i}=\left(\begin{array}{c} \begin{array}{l} \mu_{1}^{i}\\ \vdots \\ \mu_{1}^{i} \end{array}\}d\;\text{elements}\\ \begin{array}{l} \mu_{2}^{i}\\ \vdots \\ \mu_{2}^{i} \end{array}\}d\;\text{elements}\\ \vdots \\ \begin{array}{l} \mu_{m}^{i}\\ \vdots \\ \mu_{m}^{i} \end{array}\}d\;\text{elements} \end{array}\right)\in {\mathbb{R}}^{md},$$ (17)

and

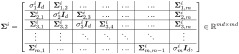
(18)
such that

$$\begin{array}{c} \Sigma_{k,l}^{i}=\sigma_{k,l}^{i}I_{d}, \end{array}$$ (19)

and $\sigma_{k,l}^{i}$ is the covariance between the *k*-th and *l*-th random vectors. In words, ***μ***^*i*^ in Eq (17) is a vector of *md* elements, consisting of the concatenation of *m* vectors. We index each of these vector by *j* = 1, …, *m*. All the entries of the *j*-th vector are equal and are set to the mean observation of the *j*-th sensor (marker) at the *i*-th point ($\mu_{j}^{i}$). In Eq (18), **Σ**^*i*^ is a matrix of size of *md* × *md*, which is the analogous concatenation of the covariance matrices of the observations. Specifically, the diagonal blocks are the diagonal matrices $\sigma_{j}^{i}I_{d}$, whose diagonal elements are the variances of the *j*-th observation, and the off-diagonal blocks are the diagonal matrices $\sigma_{k,l}^{i}I_{d}$, whose diagonal elements are the covariance between sensor (marker) *k* and sensor (marker) *l*.

**Definition 1 (empirical mean)** *Given a set* Γ *and some real function on the set* $q\in R^{\left|\Gamma \right|}$, *and a subset* Ω ⊂ Γ, *the empirical mean of **q** in* Ω *is defined by*

$$\begin{array}{c} {\left\langle q\right\rangle }_{\Omega }=\frac{1}{\left|\Omega \right|}\sum_{j\in \Omega }q\left(j\right). \end{array}$$ (20)

**Definition 2 (heterogeneity)** *Define the heterogeneity of a data point x*_*i*_ *by*

$$\begin{array}{c} h_{i}=M_{i}^{-1}\left(\Theta^{i}-{\left\langle \mu^{i}\right\rangle }_{S}1\right)\in R^{m}, \end{array}$$ (21)

*where* 〈***μ***^*i*^〉_*S*_ *is given by Definition 1*, ***M***_*i*_ *is an m* × *m diagonal matrix, whose diagonal elements are* $\mu_{j}^{i}$, *and*

$$\begin{array}{c} \Theta^{i}=[\begin{array}{c} \mu_{1}^{i}\\ \mu_{2}^{i}\\ \vdots \\ \mu_{m}^{i} \end{array}]\in R^{m}. \end{array}$$ (22)

The heterogeneity $h_{i}\in R^{m}$ captures the mutual-relationships between the expected values of the observations $\mu_{j}^{i}$ of a particular data point *x*_*i*_; if **Θ**(*j*)^*i*^ significantly deviates from *μ*^*i*^, then ***h***_*i*_(*j*) is large. Conversely, if **Θ**^*i*^(*j*) is close to *μ*^*i*^, then ***h***_*i*_(*j*) is close to zero.

**Definition 3 (weighted heterogeneity)** *Define* ***g***_*i*_ *as a weighted heterogeneity, whose jth element is given by*

$$\begin{array}{c} g_{i}\left(j\right)=\Theta^{i}\left(j\right)h_{i}\left(j\right). \end{array}$$ (23)

Under the considered statistical model, with the above definitions, the SSD ***π***_*i*_ can be written explicitly.

**Proposition 1** *The j-th element of* ***π***_*i*_ *can be approximated by*

$$\begin{array}{c} \pi_{i}\left(j\right)=\frac{1}{2m}+\frac{\epsilon -(g_{i}^{2}\left(j\right)+\sigma_{j}^{i})-2{\left\langle \Sigma_{j,\cdot }^{i}\right\rangle }_{S}+{\left\langle \Sigma^{i}\right\rangle }_{S}}{2\epsilon m-2m({\left\langle g_{i}^{2}\right\rangle }_{S}+{\left\langle \Sigma^{i}\right\rangle }_{S})}. \end{array}$$ (24)

The derivation is based on the Taylor expansion of the Gaussian function in Eq (1), where *ϵ* is the scale of the function. The proof appears in S1 Appendix.

In order to give some intuition, we consider the following special cases, where the SSD assumes a simpler form.

#### Special case 1

Suppose that the random vectors of the observation functions are independent and identically distributed, i.e., $\Sigma_{k,l}^{i}=0$ ∀*l* ∈ {1, …, *m*} and *k* ≠ *l*. In this case, the *k*-th element of ***π***_*i*_ is

$$\begin{array}{c} \pi_{i}\left(k\right)=\frac{1}{2m}+\frac{\epsilon -(g_{i}^{2}\left(k\right)+\sigma_{k}^{i})}{2\epsilon m-2m({\left\langle g_{i}^{2}\right\rangle }_{S}+{\left\langle \sigma^{i}\right\rangle }_{S})}, \end{array}$$ (25)

where

$$\begin{array}{c} \sigma^{i}=[\begin{array}{c} \sigma_{1}^{i}\\ \sigma_{2}^{i}\\ \vdots \\ \sigma_{m}^{i} \end{array}]. \end{array}$$ (26)

Note that a small value is assigned to ***π***_*i*_(*k*) if the weighted heterogeneity ***g***_*i*_(*k*) is large. In contrast, a large value is assigned to ***π***_*i*_(*k*) if ***g***_*i*_(*k*) is small. As a consequence, ***π***_*i*_(*k*) carries the heterogeneity information of the observations.

#### Special case 2

When the kernel scale *ϵ* → ∞, the information about the heterogeneity of each observation is lost, since the same weights are assigned to all the edges. As a consequence, ***π***_*i*_ becomes just a constant vector, given by

$$\begin{array}{c} \pi_{i}\underset{\epsilon \to \infty }{⟶}\frac{1}{m}1, \end{array}$$ (27)

where $1\in R^{m}$ is an all-ones vector.

### Binary hypothesis testing

Suppose that ${\left\{x_{i}\right\}}_{i=1}^{n}\in M$ are realizations of a random variable *X*, which follows a bimodal distribution stemming from two hypotheses: $H_{1}$ and $H_{2}$; $H_{1}$ has probability *α* and $H_{2}$ has probability (1 − *α*), where 0 < *α* < 1. Denote the set of data points from hypothesis $H_{1}$ by Ω_1_ and the set of data points from hypothesis $H_{2}$ by Ω_2_. Recall that for each data point *x*_*i*_, *f*_*j*_(*x*_*i*_) are the realizations of the elements of the random vector *V*^*i*^. Since *V*^*i*^ depends on the random variable *X*, assume that *V*^*i*^ also follows a bimodal distribution, which is induced by the bimodal distribution of *X*. Particularly, consider a Gaussian setting, where *V*^*i*^ is sampled from $N(m_{1},\Sigma_{1})$ with probability *α* and from $N(m_{2},\Sigma_{2})$ with probability of (1 − *α*). Similarly, assume that the random variable $V_{j}^{i}$ follows a bimodal distribution: sampled from $N(\mu_{j}^{1}1_{d},\sigma_{j}^{1}I_{d})$ with probability *α* and from $N(\mu_{j}^{2}1_{d},\sigma_{j}^{2}I_{d})$ with probability (1 − *α*), where the respective probability density functions are $f(v|\mu_{j}^{1},\sigma_{j}^{1})$ and $f(v|\mu_{j}^{2},\sigma_{j}^{2})$.

A naïve approach for binary hypothesis testing would be to directly compare the densities of the two hypotheses for each observation separately. Particularly, based on the realizations from only one observation function *f*_*j*_, the average probability of error attained in a Bayesian setting with the MAP estimator [5] is given by

$$\begin{array}{c} P_{e,j}=\alpha (1-TV(N(\mu_{j}^{1}1_{d},\sigma_{j}^{1}I_{d}),N(\mu_{j}^{2}1_{d},\sigma_{j}^{2}I_{d}))), \end{array}$$ (28)

where $TV(N(\mu_{j}^{1}1_{d},\sigma_{j}^{1}I_{d}),N(\mu_{j}^{2}1_{d},\sigma_{j}^{2}I_{d}))$ denotes the total variation between the realizations of $V_{j}^{i}|x_{i}\in \Omega_{1}$ and $V_{j}^{i}|x_{i}\in \Omega_{2}$, defined by

$$\begin{array}{c} \begin{array}{cc} TV( & N(\mu_{j}^{1}1_{d},\sigma_{j}^{1}I_{d}),N(\mu_{j}^{2}1_{d},\sigma_{j}^{2}I_{d}))=\frac{1}{2}\int_{v}|f\left(v|\mu_{j}^{1},\sigma_{j}^{1}\right)-f\left(v|\mu_{j}^{2},\sigma_{j}^{2}\right)|dv. \end{array} \end{array}$$ (29)

According to [25], consider $\frac{|\sigma_{j}^{1}-\sigma_{j}^{2}|}{\sigma (j)}\leq \frac{2}{3}$, where $\sigma \left(j\right)=\max\left\{\sigma_{j}^{1},\sigma_{j}^{2}\right\}$. The total variation above between two Gaussian distributions is bounded by

$$\begin{array}{c} TV(N(\mu_{j}^{1}1_{d},\sigma_{j}^{1}I_{d}),N(\mu_{j}^{2}1_{d},\sigma_{j}^{2}I_{d}))\leq \frac{|\mu_{j}^{1}-\mu_{j}^{2}|}{2\sqrt{\sigma (j)}}+\frac{|\sigma_{j}^{1}-\sigma_{j}^{2}|}{2\sigma (j)}. \end{array}$$ (30)

We seek another more discriminative approach for binary hypothesis testing. For this purpose, we propose a method based on the SSDs. Since the obtained SSDs represent probability distributions, the average probability of error is given by

$$\begin{array}{c} P_{e}^{\prime }=\alpha (1-TV({\left\langle \pi \right\rangle }_{\Omega_{1}},{\left\langle \pi \right\rangle }_{\Omega_{2}})). \end{array}$$ (31)

According to Proposition 1, the total variation between two SSDs associated with data points from two hypotheses can be explicitly expressed as the *l*_1_ distance given by

$$\begin{array}{c} TV({\left\langle \pi \right\rangle }_{\Omega_{1}},{\left\langle \pi \right\rangle }_{\Omega_{2}})=\sum_{k=1}^{m}\left|{\left\langle \pi \left(k\right)\right\rangle }_{\Omega_{1}}-{\left\langle \pi \left(k\right)\right\rangle }_{\Omega_{2}}\right|\\ =\sum_{k=1}^{m}|\frac{\epsilon -g_{1}^{2}\left(k\right)-\sigma_{k}^{1}-2{\left\langle \Sigma_{k,\cdot }\right\rangle }_{\Omega_{1}}+{\left\langle \Sigma \right\rangle }_{\Omega_{1}}}{2\epsilon m-2m({\left\langle g^{2}\right\rangle }_{\Omega_{1}}+{\left\langle \Sigma \right\rangle }_{\Omega_{1}})}-\frac{\epsilon -g_{2}^{2}\left(k\right)-\sigma_{k}^{2}-2{\left\langle \Sigma_{k,\cdot }\right\rangle }_{\Omega_{2}}+{\left\langle \Sigma \right\rangle }_{\Omega_{2}}}{2\epsilon m-2m({\left\langle g^{2}\right\rangle }_{\Omega_{2}}+{\left\langle \Sigma \right\rangle }_{\Omega_{2}})}|, \end{array}$$ (32)

which consists of three main components: the variances $\sigma_{k}^{1}$ and $\sigma_{k}^{2}$, the weighted heterogeneities ***g***_1_ and ***g***_2_, and the covariance **Σ**.

The total variation of the measurements in Eq (29) and the total variation between the SSDs in Eq (32) can be used to distinguish between the two hypotheses. In the following, we specify the conditions, under which the total variation based on the SSDs in Eq (31) is larger, and hence, leading to smaller error compared to the standard MAP estimator using a single observation as specified in Eq (28).

**Proposition 2** *Suppose* $\mu_{j}^{1}=\mu_{j}^{2}$ *and* $\sigma_{j}^{1}=\sigma_{j}^{2}$, *which imply by definition (or by* Eq (30)*) that the total variation between the distributions corresponding to the two hypotheses is zero, i.e*.,

$$\begin{array}{c} TV(N(\mu_{j}^{1}1_{d},\sigma_{j}^{1}I_{d}),N(\mu_{j}^{2}1_{d},\sigma_{j}^{2}I_{d}))=0. \end{array}$$ (33)

*This means that not only the standard MAP estimator but also any estimator based directly on single channel observations cannot distinguish between the two hypotheses. Conversely, from* Eq (32), *the SSDs may carry a distinction capability, that is*,

$$\begin{array}{c} \begin{array}{cc} & TV({\left\langle \pi \right\rangle }_{\Omega_{1}},{\left\langle \pi \right\rangle }_{\Omega_{2}})\\ = & \sum_{k=1}^{m}|\frac{\epsilon -g_{1}^{2}\left(k\right)-\sigma_{k}^{1}-2{\left\langle \Sigma_{k,\cdot }\right\rangle }_{\Omega_{1}}+{\left\langle \Sigma \right\rangle }_{\Omega_{1}}}{2\epsilon m-2m({\left\langle g^{2}\right\rangle }_{\Omega_{1}}+{\left\langle \Sigma \right\rangle }_{\Omega_{1}})}-\frac{\epsilon -g_{2}^{2}\left(k\right)-\sigma_{k}^{2}-2{\left\langle \Sigma_{k,\cdot }\right\rangle }_{\Omega_{2}}+{\left\langle \Sigma \right\rangle }_{\Omega_{2}}}{2\epsilon m-2m({\left\langle g^{2}\right\rangle }_{\Omega_{2}}+{\left\langle \Sigma \right\rangle }_{\Omega_{2}})}|\geq 0. \end{array} \end{array}$$ (34)

Proposition 2 demonstrates that there are cases where a single observation cannot be used for distinguishing between the two hypotheses. However, in such cases, the SSDs may enable us to distinguish the hypotheses due to possible differences in either the heterogeneity or the covariances. Proposition 2 is further demonstrated in the context of the localization toy problem in Simulation 2.

To further expand the analysis, we make the following assumptions.

**Assumption 1** *The empirical mean of the weighted heterogeneity is approximately the same under the two hypotheses*:

$${\left\langle g^{2}\right\rangle }_{\Omega_{1}}-{\left\langle g^{2}\right\rangle }_{\Omega_{2}}≃0.$$ (A.1)

**Assumption 2** *The empirical mean of the covariance matrices is approximately the same under the two hypotheses*:

$${\left\langle \Sigma \right\rangle }_{\Omega_{1}}-{\left\langle \Sigma \right\rangle }_{\Omega_{2}}≃0.$$ (A.2)

Note that if Assumptions (A.1) and (A.2) hold, implying that $2\epsilon m-2m\left({\langle g^{2}\rangle }_{\Omega_{1}}+{\langle \Sigma \rangle }_{\Omega_{1}}\right)≃2\epsilon m-2m\left({\langle g^{2}\rangle }_{\Omega_{2}}+{\langle \Sigma \rangle }_{\Omega_{2}}\right)$, then the *l*_1_ distance between the SSDs in Eq (32) can be recast as

$$\begin{array}{c} TV({\left\langle \pi \right\rangle }_{\Omega_{1}},{\left\langle \pi \right\rangle }_{\Omega_{2}})≃\sum_{k=1}^{m}|\frac{g_{1}^{2}\left(k\right)-g_{2}^{2}\left(k\right)+\sigma_{k}^{1}-\sigma_{k}^{2}+2\left({\left\langle \Sigma_{k,\cdot }\right\rangle }_{\Omega_{1}}-{\left\langle \Sigma_{k,\cdot }\right\rangle }_{\Omega_{2}}\right)}{2\epsilon m-2m({\left\langle g^{2}\right\rangle }_{\Omega_{1}}+{\left\langle \Sigma \right\rangle }_{\Omega_{1}})}|. \end{array}$$ (35)

**Proposition 3** *Suppose that Assumptions* (A.1) *and* (A.2) *hold. Suppose* $\mu_{j}^{1}=\mu_{j}^{2}$, *which implies that the upper bound of the total variation at the j-th element in* Eq (30) *only depends on the variance*

$$\begin{array}{c} TV(N(\mu_{j}^{1}1_{d},\sigma_{j}^{1}I_{d}),N(\mu_{j}^{2}1_{d},\sigma_{j}^{2}I_{d}))\leq \frac{|\sigma_{j}^{1}-\sigma_{j}^{2}|}{2\sigma (j)}. \end{array}$$ (36)

*In addition, suppose that (i) the covariance between the jth observation and the other observations under the two hypotheses is approximately equal* ${\langle \Sigma_{k,\cdot }\rangle }_{\Omega_{1}}≃{\langle \Sigma_{k,\cdot }\rangle }_{\Omega_{2}}$, *(ii) the empirical mean of the difference of variance and weighted heterogeneity of the two hypotheses is greater than the difference of j-th variance, i.e*., $\langle {\left(\sigma^{1}-\sigma^{2}\right)}^{⊤}\left(g_{1}^{2}-g_{2}^{2}\right)\rangle \geq \left|\sigma_{j}^{1}-\sigma_{j}^{2}\right|$, *and (iii) the weighted heterogeneity of* $H_{1}$ *is sufficiently large such that* ${\langle g^{2}\rangle }_{\Omega_{1}}\geq \epsilon -{\langle \Sigma \rangle }_{\Omega_{1}}-\sigma \left(j\right)$. *Then, the l*_1_ *distance between the SSDs can be recast and bounded from below by*

$$\begin{array}{c} \begin{array}{cc} TV({\left\langle \pi \right\rangle }_{\Omega_{1}},{\left\langle \pi \right\rangle }_{\Omega_{2}}) & ≃\sum_{k=1}^{m}|\frac{g_{1}^{2}\left(k\right)-g_{2}^{2}\left(k\right)+\sigma_{k}^{1}-\sigma_{k}^{2}}{2\epsilon m-2m({\left\langle g^{2}\right\rangle }_{\Omega_{1}}+{\left\langle \Sigma \right\rangle }_{\Omega_{1}})}|\\ & \geq \frac{\left\langle {\left(\sigma^{1}-\sigma^{2}\right)}^{⊤}\left(g_{1}^{2}-g_{2}^{2}\right)\right\rangle }{2\sigma (j)}\geq \frac{|\sigma_{j}^{1}-\sigma_{j}^{2}|}{2\sigma (j)}. \end{array} \end{array}$$ (37)

*It follows that*

$$\begin{array}{c} TV(N(\mu_{j}^{1}1_{d},\sigma_{j}^{1}I_{d}),N(\mu_{j}^{2}1_{d},\sigma_{j}^{2}I_{d}))\leq TV({\left\langle \pi \right\rangle }_{\Omega_{1}},{\left\langle \pi \right\rangle }_{\Omega_{2}}). \end{array}$$ (38)

This proposition implies that when the assumptions hold, the probability of error based on SSD, which indirectly takes into account the mutual-relations between all observations, facilitates a better distinction of the two hypotheses compared to the standard MAP estimator computed from the best sensor. This property is further demonstrated in the localization toy problem in Simulation 3.

**Proposition 4** *Suppose the conditions of Proposition 3 hold. In addition, suppose that the random vectors of the observations are independent and identically distributed, i.e*., $\sigma_{k,j}^{1}=\sigma_{k,j}^{2}=0$ ∀*k* ≠ *l*, *then*

$$\begin{array}{c} TV(N\left(m_{1},\Sigma_{1}\right),N\left(m_{2},\Sigma_{2}\right))\leq TV({\left\langle \pi \right\rangle }_{\Omega_{1}},{\left\langle \pi \right\rangle }_{\Omega_{2}}). \end{array}$$ (39)

This proposition shows that the SSDs enable us a better distinction between the two hypotheses compared to the MAP estimator based on the distributions of the multi-feature observations. In other words, the heterogeneity comprising the SSD has a significant contribution to the ability to recover the information about the latent data points ${\left\{x_{i}\right\}}_{i=1}^{n}$ on the underlying manifold M, thereby leading to accurate binary hypothesis testing.

### Imaging mass cytometry (IMC)

IMC is a relatively new imaging method, which enables to examine tumors and tissues at subcellular resolutions, giving rise to images consisting of the intensities of multiple proteins [13–15]. This acquisition platform, combined with computational methods, has recently been the subject of many studies. Various image processing and analysis techniques for IMC datasets can be found in [26], where it is shown that single-cell segmentation can be accomplished successfully with supervised classifiers, leading to the characterization of cell co-occurrence and cell composition of different types of tissues and samples. In [27], an IMC dataset with 37 markers is used for cell segmentation and cell clustering based on random 125 × 125*μm*^2^ patches collected from breast cancer patients. This dataset is jointly analyzed with multi-platform genomics data, where it is shown that classifiers can be iteratively trained in a supervised manner to learn from the IMC pixels the corresponding cell expression levels. In [14], spanning-tree progression analysis combined with samples’ type provided by pathologists is used for cell population and cell transition identification. In contrast to these methods, our approach focuses on extracting the mutual-relationships between markers at likely tumor cells regions at large, circumventing cell segmentation.

In this work, our goal is to identify the sensitivity of lung cancer subjects to treatment with PD-1 axis blockers, given their IMC multiplexed observations. More specifically, we aim at a binary prediction task: identifying whether the subjects responded or did not respond to the treatment. We analyze two IMC datasets consisting of baseline/treatment tumor samples from non-small cell lung cancer subjects profiled with 29 markers, representing phenotype and functional properties of both tumor and immune cells. The markers are denoted by LipoR, VIM, T-BET, CD47, Cytokeratin, CD45RO, PD-L1, GAPDH, B7-H3, LAG-3, TIM-3, FOXP3, CD4, B7-H4, CD68, PD1, CD20, CD8, CD25, VISTA, KI-67, B2M, CD3, IDO-1, PD-L2, GZB, Histone 3, DNA1 and DNA2. The resolution of the IMC images is 1 *μm*^2^ per pixel. The first dataset, denoted Dataset 1, consists of 55 subjects (samples), and the second dataset, denoted Dataset 2, consists of 29 subjects (samples). These subjects received treatment with PD-1 axis blockers. Based on the clinical sensitivity to the treatment, the subjects are categorized as: durable clinical benefit (denoted with the label responders) and no durable benefit (denoted with the label non-responders).

Prior to our analysis, a standard pre-processing using z-score normalization is applied to each marker. We remark that the mean and the standard deviation are computed based only on pixels in which the marker has non-zero values. In addition, we apply a 3 × 3 median filter to every image in Dataset 2. We note that the median filter is not applied to Dataset 1, because the typical expression levels in this dataset are sparse, and in this situation, application of a filter may destroy the signal. This is demonstrated in S1 Fig, where we apply the median filter to the expression levels of few important markers and observe a degenerate result. The markers exhibiting such sparse expression levels in Dataset 1 are VIM, T-BET, CD45RO, PD-L1, GAPDH, B7-H3, LAG-3, FOXP3, B7-H4, PD1, CD20, CD8, CD25, VISTA, KI-67, CD3, PD-L2 and GZB. Conversely, in Dataset 2, the typical expression levels are dense, and as a result, the median filter enhances the content of the images.

Our analysis does not consider the entire image, but rather focuses on ROIs located at the highest Cytokeratin expression levels, as Cytokeratin is expressed only in the tumor cells. As noted above, this circumvents cell segmentation that is typically required in other analyses techniques. At each ROI, we consider a “stack” of *m* image patches from all the *m* markers, where each patch consists of *d* = *b* × *b* pixels. We select *N* ROIs per sample by searching the patches with the maximal mean value of Cytokeratin. This is implemented by a 2D convolution applied to the Cytokeratin image with a constant kernel of size *b* × *b*. We deliberately avoid patch overlap by assigning zero values to the pixels of each ROI, once it is selected.

**Fig 2:**
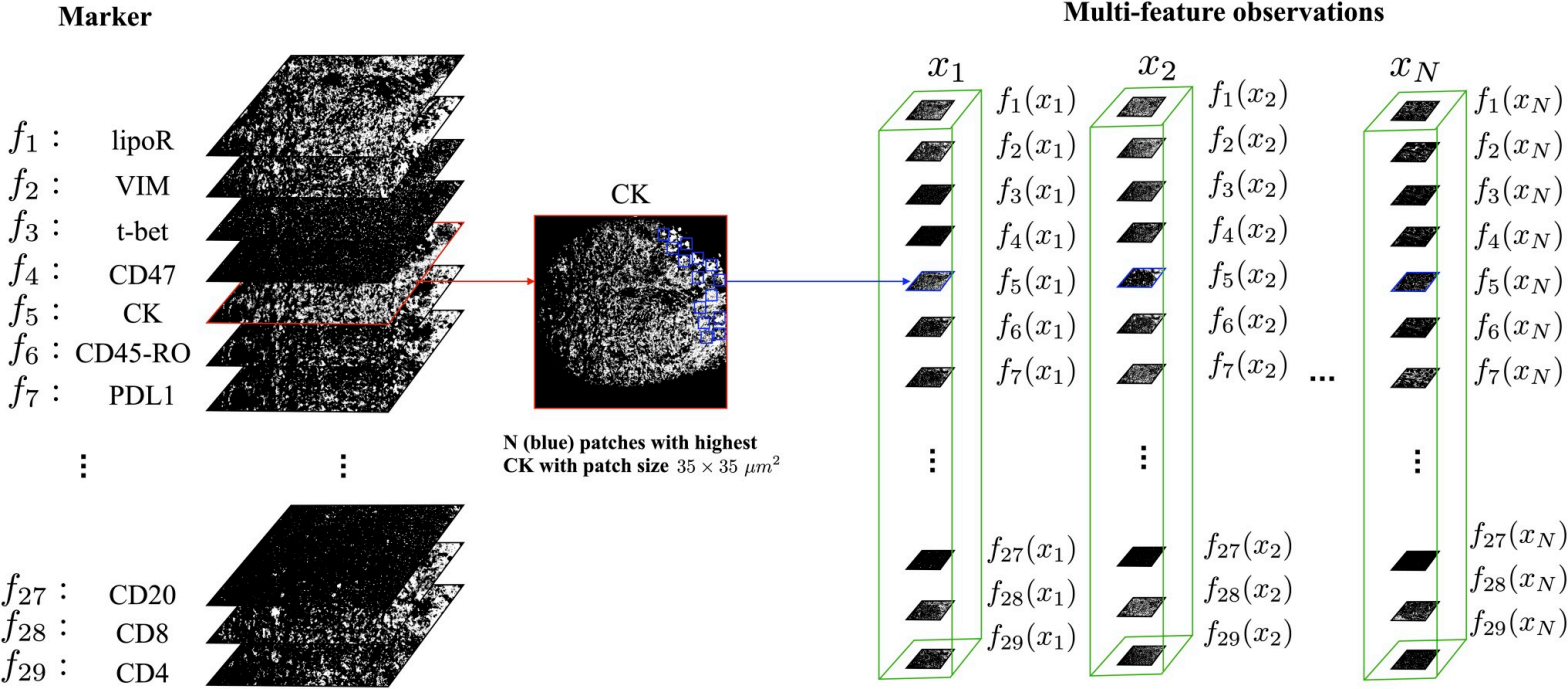
An illustration of the IMC multi-feature observations from a single subject. In the IMC data of a single subject, we focus first on the Cytokeratin marker, which is used as an indicator of tumor cells. We select *N* ROIs located at the highest Cytokeratin expression levels. These ROIs are patches of size *b* × *b* *μm*^2^, where here *b* set to 35. We assume that these ROIs have intrinsic hidden states represented by *x*_*i*_. The expression of all 29 markers at these ROIs are viewed as the multi-feature observations ${\left\{f_{j}\left(x_{i}\right)\right\}}_{j=1}^{29}$ for *i* = 1, …, *N*.

Using the present work notation, the intrinsic representation of the information embodied at each ROI is denoted by *x*_*i*_, which is assumed to be a data point from some hidden manifold M. Our working hypothesis is that the distribution of ${\left\{x_{i}\right\}}_{i=1}^{n}$ on the hidden manifold M is bimodal, which is induced by the sensitivity of the subjects to the treatment; data points *x*_*i*_ at ROIs within tissues of responders are located in one region of the manifold, and data points *x*_*i*_ at ROIs within tissues from non-responders are located in another region of the manifold. Given a data point *x*_*i*_, our two hypotheses, $H_{\text{r}}$ and $H_{\text{n}}$, are whether *x*_*i*_ is a realization from the distribution of responders or non-responders, respectively. Here, *n* = *N* × *P* is the total number of ROIs across all the samples, where *P* is the number of samples and *N* is the number of ROIs we consider in each sample. Next, recall that the intrinsic representation *x*_*i*_ is hidden. Instead, the accessible observations are the expression levels of the markers at the ROIs, which are represented mathematically by the observation functions $f_{j}\left(x_{i}\right)\in R^{d}$ for *j* = 1, …, *m*, where *m* = 29 is the number of markers. The domain of the observation functions is the hidden intrinsic manifold M and the range is of dimension *d* = *b* × *b*, which is the size of a patch of an image of one marker. Namely, the values assigned by *f*_*j*_(*x*_*i*_) to *x*_*i*_ correspond to the expression level of marker *j* at the ROI associated with *x*_*i*_. The set of patches of all the markers at a specific ROI *x*_*i*_ is a set of multi-feature observations ${\left\{f_{j}\left(x_{i}\right)\right\}}_{j=1}^{29}$. In Fig 2, we illustrate the IMC multiplexed observations from a single subject and the set up under consideration.

The identification of the subject’s response to treatment from the IMC data is based on the application of the proposed method described in Box 1. This algorithm fits well the problem at hand due to the following main reasons. First, directly comparing observations from the different markers, *f*_*j*_(*x*_*i*_), is inapplicable since each ROI comprises different cells and different tissue structures. Our method circumvents this problem by computing an intrinsic signature of the observations. Second, due to the different dynamics of the nominal values of the observations from the different markers, a naïve concatenation of the multi-feature observations is inadequate (in contrast to the localization example).

Before applying the proposed method, we test empirically that Assumptions (A.1) and (A.2) hold. Note that for this test only, the true response status is used. To test Assumption (A.1), we compute

$$\frac{\left|\right|{\left\langle g^{2}\right\rangle }_{\Omega_{r}}-{\left\langle g^{2}\right\rangle }_{\Omega_{n}}{\left|\right|}_{2}}{\sqrt{\left|\right|{\left\langle g^{2}\right\rangle }_{\Omega_{r}}{\left|\right|}_{2}\cdot \left|\right|{\left\langle g^{2}\right\rangle }_{\Omega_{n}}{\left|\right|}_{2}}}$$

and to test Assumption (A.2) we compute

$$\frac{{||\left\langle \Sigma \right\rangle }_{\Omega_{r}}-{\left\langle \Sigma \right\rangle }_{\Omega_{n}}{\left|\right|}_{F}}{\sqrt{{||\left\langle \Sigma \right\rangle }_{\Omega_{r}}{\left|\right|}_{F}\cdot {||\left\langle \Sigma \right\rangle }_{\Omega_{n}}{\left|\right|}_{F}}},$$

where Ω_*r*_ and Ω_*n*_ denote the sets of responders and non-responders, respectively. The respective values we obtain for Dataset 1 are 0.12 and 0.08 and for Dataset 2 the values for responders and non-responders are 0.03 and 0.04. This implies that the conditions in the two assumptions are approximately satisfied.

We analyze the two datasets separately, since the datasets were collected around a year apart, and internal acquisition system parameters were modified during that time. We apply the proposed method presented in Box 1 to the observations, ${\left\{f_{j}\left(x_{i}\right)\right\}}_{j=1}^{29}$ for *i* = 1, …, *n*, resulting in a low-dimensional representation of the ROIs. Then, we apply to the low-dimensional representation an RBF SVM classifier with a leave-one-subject-out (LOSO) cross-validation [5] in order to predict the response to treatment. In order to assess the prediction performance for each subject, we compute the average of the prediction results of all the ROIs of that subject. We note that the prediction is based on features computed per patch (rather than per subject). Therefore, in practice, the number of samples used for the cross-validation is 55 × *N* for Dataset 1 and 29 × *N* for Dataset 2. Importantly, at each cross-validation iteration, all *N* patches of a subject were removed from the training set, and were only used for testing the classifier.

We compare the SSD-based representation obtained by the propose method to other representations obtained by three competing algorithms. The first is a direct application of diffusion maps to the sets of multi-feature observations ${\left\{f_{j}\left(x_{i}\right)\right\}}_{j=1}^{29}$ for *i* = 1, …, *n*. The second and the third are based on HKS [16] and WKS [17], respectively, replacing the SSD as the features of each ROI at the first stage of the proposed method in Box 1. The dimensions of the representation obtained by the proposed method and by the three competing algorithms are determined by a variant of the Jackstraw method, described in Determining the dimension of data.

**Fig 3:**
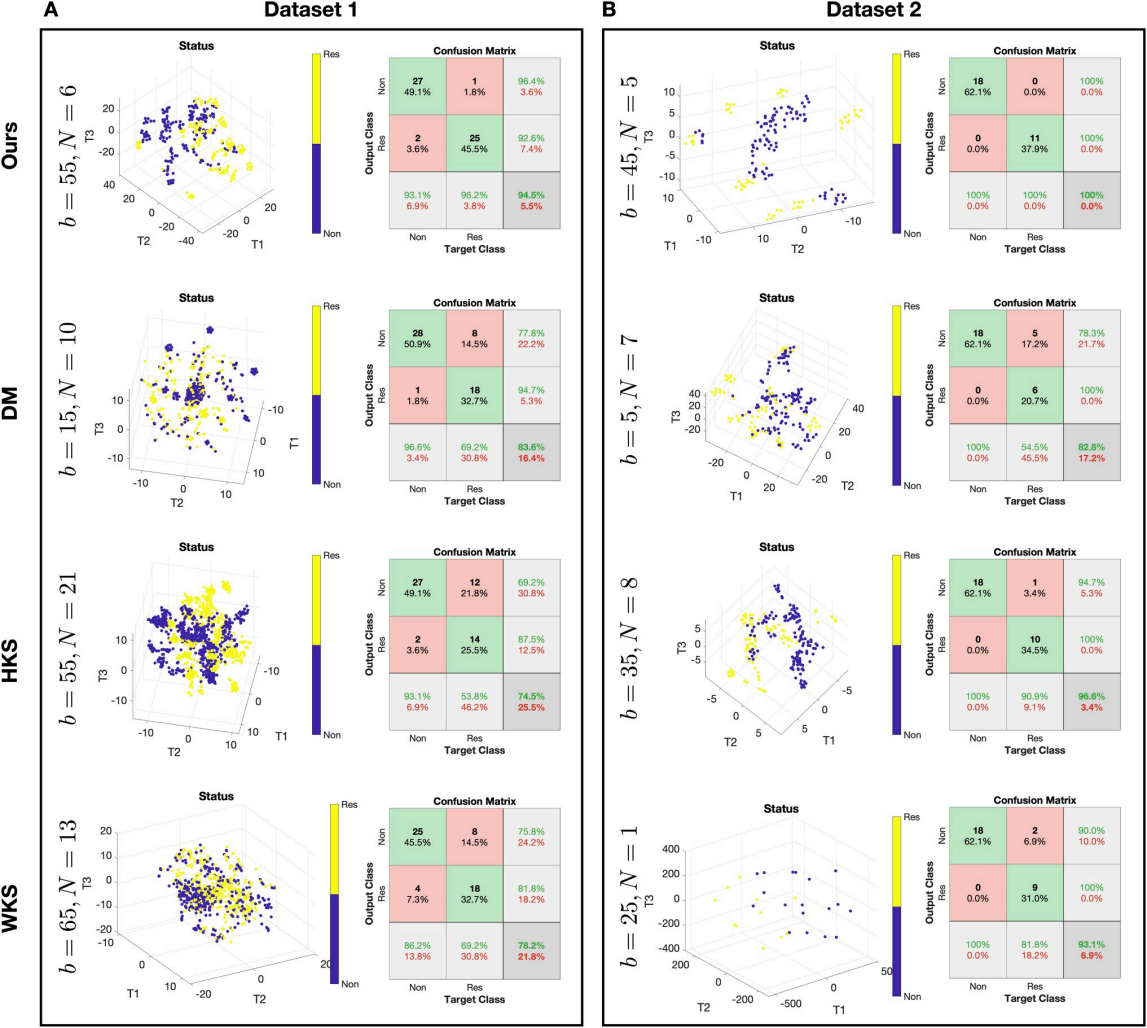
Treatment response predictions. (A) Prediction for Dataset 1. (B) Prediction for Dataset 2. In each panel, at each row, we plot the best algorithm configuration, the 3D t-SNE visualization of the patches representation colored by the response status, and the confusion matrix of the response prediction obtained by a leave-one-subject-out cross-validation using an RBF SVM classifier. The rows present the results of the different methods.

Fig 3 presents the predictions of treatment response by the SSD, HKS, WKS based algorithms as well as DM. We show the 3D t-SNE visualization [28] of the patch representation obtained as an output of the different algorithms and the confusion matrix of the prediction obtained by an RBF SVM classifier applied to the patch representation. To complement the results, in Table 1, we present the area under the ROC curve (AUC) of the treatment response predictions. We note that the AUC obtained for Dataset 2 without the median filter pre-processing is 0.931. For each algorithm, the presented prediction results are based on the patch size and number of patches configuration that yielded the best empirical performance using cross-validation as described above. The best configuration (patch size *b* × *b* and number of patches per subject *N*) of each algorithm is presented in Fig 3 on the left.

**Table 1:** ROC AUC for predictions of treatment sensitivity.

	Proposed Method	DM	HKS	WKS
Dataset 1	0.9455	0.8364	0.7455	0.7818
Dataset 2	1	0.8276	0.9655	0.9310

In the t-SNE plots, we observe that the unsupervised separation of the patches from responders and non-responders is most pronounced in the low-dimensional representation obtained by the proposed method. This distinct visual separation, which was obtained by the proposed algorithm without access to any response outcome information. The color in the figures indicates the response status. Note that the embedding is obtained by the unsupervised algorithm and the color labels are overlaid to demonstrate the degree of separability between patches from responders and non-responders, which implies that this patch representation is informative and useful for the subsequent response prediction. This result, which is obtained in an unsupervised manner, distinguishes the current work from the computational methods for IMC data described above that rely on supervised analysis. Indeed, we observe that the prediction accuracy obtained based on the proposed method is superior compared to the other three competing methods. We note that Dataset 1 consists of 26 responders and 29 non-responders, so that the chance level of accurate prediction of a subject with durable clinical benefit is 47.27%, and Dataset 2 consists of 11 responders and 18 non-responders, thus, the chance level of accurate prediction is 37.93%.

The derivations in Theoretical analysis imply that the capability to distinguish between the two hypotheses (sensitivity or insensitivity to treatment) highly depends on the mutual relationships between the markers, which we explicitly define and term heterogeneity (Definition 2). Since our empirical study demonstrates that our approach facilitates a distinct separation between ROIs of subjects according to their treatment response, by the theoretical analysis, we conclude that the heterogeneity between the markers is where the information about the sensitivity to treatment lies.

We examine the sensitivity of the tested algorithms to the choice of the hyperparameters: the number of ROIs per subject *N* and the size of the patch *b* × *b*. In S2 Fig, we plot heatmaps of the treatment prediction accuracy obtained based on different choices of hyperparameters. We observe that within a relatively wide range of parameter values, the performance of the proposed method in Box 1 is high and insensitive to the particular choice of parameters. In addition, we note that the range of patch size where high performance is attained is centered at size 45 × 45 *μm*^2^. As the size of tumor cells vary within a range of 10–30 *μm*^2^ (a mean of 20 *μm*^2^), and the size of a lymphocyte ranges from 8–12 *μm*^2^ (a mean of 10 *μm*^2^), it implies that high performance is attained when patches are likely including more than one cell and cell type. Conversely, we observe that the competing methods do not show the same degree of robustness to the choice of hyperparameters, and good performance is attained only for very particular (isolated) parameter values.

To further exploit the robustness of the proposed method, we implemented an ensemble of the classifiers based on different values of hyperparameters. The implementation of the combination is based on [29]. The performance of the combined classifiers is presented in S3 Fig. We observe that the prediction accuracy of this ensemble is comparable to the prediction accuracy obtained based on the classifier with the best parameter configuration, demonstrating that the particular choice of hyperparameters can be circumvented.

Our premise is that the resulting high prediction accuracy is attributed mainly to the unsupervised informative representation obtained by our approach, rather than the classifier type. To support this claim, we repeat the analysis by replacing the RBF SVM classifier with Random Forest [5]. The results are shown in S4 Fig and demonstrate comparable performance and similar trends.

To show that Stage 2 of the proposed method in Box 1 is essential, we plot in S5 Fig heatmaps of the multi-feature observations and the SSD features resulting from Stage 1 of the algorithm. The heatmaps of the multi-feature observations demonstrate that there is no obvious difference between responders and non-responders, implying that the response status prediction is a non-trivial task. In addition, inspection of the heatmaps with the SSD features does not reveal apparent distinction between responders and non-responders. Recalling that after Stage 2, as presented in the t-SNE plots in Fig 3, this distinction becomes evident, demonstrating the contribution of Stage 2.

### Discussion

We presented a two-step graph analysis approach. The first step is applied to the multi-feature observations of the data points, where their mutual-relationships are extracted. This step is implemented by computing the SSD of a random walk defined on the graph whose nodes are the observations. The resulting SSD can be viewed as a signature or a characteristic vector of the data point and is analogous to traditional signatures from other domains, such as the heat kernel signature (HKS) [16] or the wave kernel signature (WKS) [17] in the field of shape analysis, and PageRank [30] in web page ranking (see Related work). The second step is applied to the signatures obtained at the first step, for the purpose of constructing an intrinsic low-dimensional representation of all the data points.

Previous attempts to analyze such mulitplexed datasets involve various approaches, including direct comparisons of the marker expressions, the cell morphology, and interactions in cell neighborhoods, to name but a few [26]. Our method introduces a new approach, building a new representation of the multiplexed data in two steps. Since each of the steps involves a construction of a graph, the entire procedure can be viewed as building a graph of graphs.

While the algorithm is described in a general setting of multiplexed data fusion, our theoretical analysis is focused on binary hypothesis testing. In comparison with a traditional statistical estimation approach, we show that our method exhibits advantages, implying that the mutual-relationships between the multi-feature observations are well captured. In the context of IMC, this could minimize the effect of deviation in individual marker scores and cell/tissue heterogenenity.

We apply the proposed method to two IMC datasets and show that solely from the imaging data, we can distinguish between two different sensitivity levels to treatment. Since our approach does not rely on rigid prior knowledge or access to labels, it has the potential of identifying biological relevance of novel parameters or marker patterns in treatment responses by analyzing dominant factors contributing more to the model stratification. Importantly, we remark that in contrast to common practice, the proposed approach does not require cell-segmentation as a precursor.

In addition to the demonstrated advantage of the proposed method over the competing methods in terms of superior prediction accuracy of treatment response, the proposed method is also more computationally efficient. In S6 Fig, we present the run time of the two stages of the proposed method in Box 1 and compare them to the run time of the three competing methods. We note that the HKS and WKS replace the proposed SSD in Stage 1, but, their respective Stage 2 are similar. In Stage 1, we observe that the proposed method in Box 1 is faster than the competing methods. The main difference between the algorithms in Stage 1 is due to the fact that the SSD is proportional to the degree of the local graph, and as a result, it can be computed efficiently from the degree vector. Conversely, both HKS and WKS require eigenvalue decomposition, which is computationally more demanding. In addition, the direct application of DM just involves Stage 2 of the proposed algorithm. Seemingly, the run time of the algorithms in Stage 2 should have been the same, yet, we observe that DM is slower. This difference is attributed to the significantly different size of the feature space. In DM, the multi-feature observations are simply concatenated, giving rise to feature space (input of Stage 2) of size (*b* × *b* × *m*) × (*P* × *N*), where *b* × *b* is the size of patch, *m* is the number of biomarkers, and *P* is the number of subjects. Conversely, in Stage 2 of Algorithm 1, HKS, and WKS, the feature space (input of Stage 2) is only of size *m* × (*P* × *N*).

It is conceivable that the most important hyperparameter of our method is the scale parameter. In S7 Fig, we present a toy example demonstrating that different values of the scale parameter *ϵ* lead to multiscale signatures capturing local and global features. We demonstrate that different scales facilitate the extraction of different features of the data. In future work, we plan to further explore the role of the scale and to devise multiscale signatures. Another possible direction for future research relies on the fact that our method is general and can be extended to other multiplexed datasets. For example, hyper spectral imaging, sensor networks, spatial multiplexed proteomics, and spatial transcriptomics assays is a representative subset of distinct technologies from diverse domains of science and engineering that share common data structures. The data in all these modalities consist of high-dimensional multivariate observations (*m*-dimensional feature space) collected at different spatial positions, and therefore, can be analyzed using similar computational methodologies. Furthermore, in many studies practitioners collect datasets consisting of multiple spatial assays of this type, each capturing such data from a single biological sample, patient, or hyper spectral image, etc. Each of these spatial assays could be characterized by several regions of interest (ROIs), giving rise to a setting similar to the IMC problem considered here. Specifically, we plan to examine applications of the proposed graph of graphs analysis to spatial transcriptomics such as Slide-seq [31], High-Density Spatial Transcriptomics [32, 33], MIBI-TOF [34], and DBiT-seq [35].

## Materials and methods

### Diffusion maps

Manifold learning is a class of nonlinear techniques that embeds high dimensional data points into a low dimensional space, relying on the assumption that the high-dimensional data lie on a low dimensional manifold M [2–4]. In order to “learn” the manifold from a discrete set of data points, a graph is typically defined, where the graph nodes are the data points and the edges are determined according to some similarity notion. Since the manifold information is entirely captured by its Laplacian, the discrete counterpart, the graph Laplacian is used to build a low-dimensional embedding that respects the manifold in some sense [36]. To this end, common practice is to compute and exploit the spectral decomposition of the graph Laplacian. Diffusion maps is one of these methods, which constructs a random walk on the graph and represents the data points in a low-dimensional space preserving the neighborhood information [4].

Consider a set of data points ${\left\{y_{i}\right\}}_{i=1}^{b}$, where $y_{i}\in R^{d}$ for *i* = 1, …, *b*. An undirected weighted graph G=(V,E,W) is constructed from the data points, where the vertex set is $V=(y_{1},y_{2},...,y_{b})$ and the weights of the edges connecting two vertices are determined by a measure of similarity between any two data points, e.g., by

$$\begin{array}{c} W\left(i,j\right)=\exp(-\frac{\left|\right|y_{i}-y_{j}{\left|\right|}_{2}^{2}}{2\epsilon }), \end{array}$$ (40)

where *i*, *j* ∈ {1, …, *b*} and *ϵ* > 0 is a scale parameter. Common practice is to set *ϵ* as the median of the distances between the graph nodes. Note that *ϵ* implicitly induces a notion of locality: it can be viewed as the (squared) radius of the neighborhood around each node, so that only nodes within this radius are considered as neighbors in the graph.

Next, a random walk ***P*** on the data points is constructed by normalizing the weight matrix ***W***

$$\begin{array}{c} P\left(i,j\right)=\frac{W(i,j)}{d(i)}, \end{array}$$ (41)

where $d\left(i\right)=\sum_{j=1}^{n}W\left(i,j\right)$. ***P*** is the transition matrix of a Markov chain defined on the data points ${\left\{y_{i}\right\}}_{i=1}^{b}$ (graph vertexes), where the entry *p*(*i*, *j*) describes the probability of a random walk transitioning from the node *y*_*i*_ to the node *y*_*j*_ in a single step. Raising the transition matrix ***P*** to a power *t* can be viewed as applying the Markov chain to the data points *t* times.

Since ***P*** is similar to a symmetric and positive-define matrix, ***P*** has a biorthogonal right- and left-eigenvectors ${\left\{\varphi_{i},\upsilon_{i}\right\}}_{i=1}^{b}$ with the eigenvalues 1 = λ_1_ ≥ λ_2_ ≥ … ≥ λ_*b*_ ≥ 0. Consequently, the spectral decomposition of ***P***^*t*^ is given by

$$\begin{array}{c} P_{t}\left(i,j\right)=\sum_{k=1}^{b}\lambda_{k}^{t}\varphi_{k}\left(i\right)\upsilon_{k}\left(j\right). \end{array}$$ (42)

The diffusion distance $D_{t}^{2}\left(i,j\right)$ between two data points *y*_*i*_ and *y*_*j*_ in the data set is defined by

$$\begin{array}{c} D_{t}^{2}\left(i,j\right)=\sum_{k=1}^{b}\frac{{\left(P_{t}\left(i,k\right)-P_{t}\left(j,k\right)\right)}^{2}}{\upsilon_{1}\left(k\right)}, \end{array}$$ (43)

which measures the similarity of two points based on the evolution of their probability distributions, and depends on all possible paths of length *t* in the graph between any two points. Namely, if two points are connected by a large number of paths, then the diffusion distance between them will be small. Conversely, if there are only few paths connecting two points, then the diffusion distance between them will be large.

The diffusion maps is defined by [4]

$$\begin{array}{c} \Phi_{t}:y_{i}\mapsto {\left(\lambda_{2}^{t}\varphi_{2}\left(y_{i}\right),\lambda_{3}^{t}\varphi_{3}\left(y_{i}\right),...,\lambda_{l}^{t}\varphi_{l}\left(y_{i}\right)\right)}^{⊤}. \end{array}$$ (44)

We remark that in many cases, due to the typical fast decay of the eigenvalues of ***P***^*t*^, *l* can be set to be smaller than *d*, thereby achieving dimension reduction. In addition, ***φ***_1_ is a constant vector and therefore is not used in the mapping.

It can be shown that the diffusion distance can be approximated by the eigenvalues and eigenvectors by [4]

$$\begin{array}{c} D_{t}^{2}\left(i,j\right)=\sum_{k=1}^{b}\lambda_{k}^{2t}{\left(\varphi_{k}\left(i\right)-\varphi_{k}\left(j\right)\right)}^{2}\approx \left|\right|\Phi_{t}\left(i\right)-\Phi_{t}\left(j\right){\left|\right|}^{2}, \end{array}$$ (45)

where equality is reached for *l* = *b*. Namely, the diffusion distance can be approximated by the Euclidean distance between the diffusion maps of the data points.

### Determining the dimension of data

A common problem in diffusion maps setting is how to choose the dimension *l*. The authors in [6] proposed *Jackstraw* to identify the number of principal components (PCs) in the context of principal component analysis (PCA) [37]. We present here a variant of *Jackstraw*, adapting it to diffusion maps.

Given a random walk ***P*** constructed from a set of *b* data points, the associated eigenvalues are 1 = λ_1_ ≥ λ_2_ ≥ … ≥ λ_*b*_ with corresponding right-eigenvectors ${\left\{\varphi_{i}\right\}}_{i=1}^{b}$. Collect the eigenvalues into a vector, denoted by **λ**, where **λ** = (λ_1_, λ_2_, …, λ_*b*_)^⊤^. Let $P_{k}^{*}$ consist of the random permutation of ***P***. Apply eigenvalue decomposition to $P_{k}^{*}$ and obtain the corresponding eigenvalues vector $\lambda_{k}^{*}$. Repeat this shuffling procedure *s* times and obtain a set of vectors ${\left\{\lambda_{k}^{*}\right\}}_{k=1}^{s}$. The dimension of the representations is determined by

$$\begin{array}{c} l=\underset{x=\{1,...,b\}}{\text{arg}\;\text{min}}(\lambda \left(x\right)\geq \max\{|\lambda_{k}^{*}\left(x\right)|\}),\;\forall k=\left\{1,...,s\right\}. \end{array}$$ (46)

Note that the absolute values of the eigenvalues of $P_{k}^{*}$ are considered because $P_{k}^{*}$ is not necessarily symmetric and therefore its eigenvalues $\lambda_{k}^{*}$ are not guaranteed to be real.

### Related work

#### Heat kernel signature

There are several shape analysis signatures obtained by spectral methods with different geometric properties such as isometry and deformation invariance [16, 17, 38, 39], related to the proposed method. One of the notable shape signatures is based on the heat diffusion on a shape, called Heat Kernel Signature (HKS) [16]. Broadly, the HKS is obtained by the eigenvalue decomposition of the heat kernel defined on the shape. In the context of our problem, since the heat kernel and the Laplace-Beltrami operator Δ_*i*_ share the same eigenbasis, and since the discrete graph Laplacian converges (point-wise) to the Laplace-Beltrami [40]

$$\begin{array}{c} \frac{1}{\epsilon }L_{i}=\frac{I-P_{i}}{\epsilon }\overset{N\to \infty }{\underset{\epsilon \to 0}{\to }}\Delta_{i}, \end{array}$$ (47)

then, a discrete counterpart of HKS is given by

$$\begin{array}{c} x_{i}\mapsto [{\text{HKS}}_{t}(f_{1}\left(x_{i}\right)),{\text{HKS}}_{t}(f_{2}\left(x_{i}\right)),\ldots ,{\text{HKS}}_{t}(f_{m}\left(x_{i}\right))], \end{array}$$ (48)

where

$$\begin{array}{c} {\text{HKS}}_{t}(f_{j}\left(x_{i}\right))=\sum_{k=1}^{m}\exp\left(-\left(1-\lambda_{k}\right)t\right)\psi_{k}^{2}\left(j\right), \end{array}$$ (49)

λ_*k*_ and ***ψ***_*k*_ are the *k*-th eigenvalue and *k*-th eigenvector of the random walk, respectively, and *t* is the number of random walk steps on the graph. For more details on HKS, we refer the readers to [16].

Similarly to the DKS in Eq (12), the HKS can also be viewed as a low-pass filter. Observe that, for small *t*, the HKS approximates the DKS by Taylor expansion; for other *t* values, the weights assigned by the HKS decay faster than the weights of DKS, and therefore, the DKS gives more attention to finer structures.

#### Wave kernel signature

Another related shape signature is built by the wave function to the Schrödinger equation describing the quantum mechanical particles, called Wave Kernel Signature (WKS) [17]. Similarly to the HKS, the WKS is given by

$$\begin{array}{c} x_{i}\mapsto [{\text{WKS}}_{t}(f_{1}\left(x_{i}\right)),{\text{WKS}}_{t}(f_{2}\left(x_{i}\right)),\ldots ,{\text{WKS}}_{t}(f_{m}\left(x_{i}\right))], \end{array}$$ (50)

where

$$\begin{array}{c} {\text{WKS}}_{t}(f_{j}\left(x_{i}\right))=\sum_{k=1}^{m}C_{t}\exp(-\frac{{\left(\log t-\log\left(1-\lambda_{k}\right)\right)}^{2}}{2\sigma^{2}})\psi_{k}^{2}\left(j\right), \end{array}$$ (51)

with

$$\begin{array}{c} C_{t}={\left(\sum_{k=1}^{m}\exp(-\frac{{\left(\log t-\log\left(1-\lambda_{k}\right)\right)}^{2}}{2\sigma^{2}})\right)}^{-1}, \end{array}$$ (52)

λ_*k*_ and ***ψ***_*k*_ are the *k*-th eigenvalue and *k*-th eigenvector of the random walk, and *t* is number of random walk steps on the graph. While the DKS and HKS are viewed as lowpass filters, we note that the WKS can be viewed as a band-pass filter. For more details, we refer the readers to [17].

#### Nodes ranking

In the context of web page ranking, there are several traditional algorithms based on the spectral analysis of directed graphs. Among them, the celebrated PageRank score is based on the stationary distribution of a random walk representing the popularity of linked web pages [30]. Hyper induced topic search (HITS) is another related algorithm, identifying the influential nodes using a random walk on a graph. There, the graph nodes are the web pages, which are divided into two groups: authorities and hubs [41]. Both algorithms address the problem of web page ranking, where PageRank depends on the incoming links whereas HITS focuses on the outgoing links.

### Localization toy problem

To illustrate the challenge in the problem setting and the generality of the proposed solution, we present three simulations of different localization problems. The Matlab code is available in S1 Matlab Code.

#### Simulation 1

Consider 800 objects on a 2-sphere in $R^{3}$ that can be located at four different regions. Each region consists of 200 objects. The positions of the objects are measured by 5 sensors, giving rise to the following set of observations ${\left\{f_{j}(x_{i})\right\}}_{j=1}^{5}\in R^{100}$, where *j* is the index of the sensor and *i* is the index of the object (position). Each sensor measures the position in *d* = 100 coordinates in the following way

$$\begin{array}{c} R^{100}∋f_{j}\left(x_{i}\right)∼N\left(0,\sigma_{i}^{2}I_{100}\right), \end{array}$$ (53)

where the standard deviation of the measurement depends on the distance between the position of the sensor and the position of the object $\sigma_{i}=20\exp(-∥x_{i}-s_{j}{∥}_{2}^{2})$, ***I***_100_ is the identity matrix of size 100 × 100, ‖ ⋅ ‖_2_ denotes the Euclidean norm and *s*_*j*_ denotes the 3D position of sensor *j* ∈ {1, …, 5}. In other words, each object position is captured by *d* = 100 realizations of a Gaussian random variable with variance that is proportional to the distance between the sensor position and the object position. Note that the positions are captured by the sensor through the variance, therefore, they are difficult to infer directly by the multi-feature observations.

**Fig 4:**
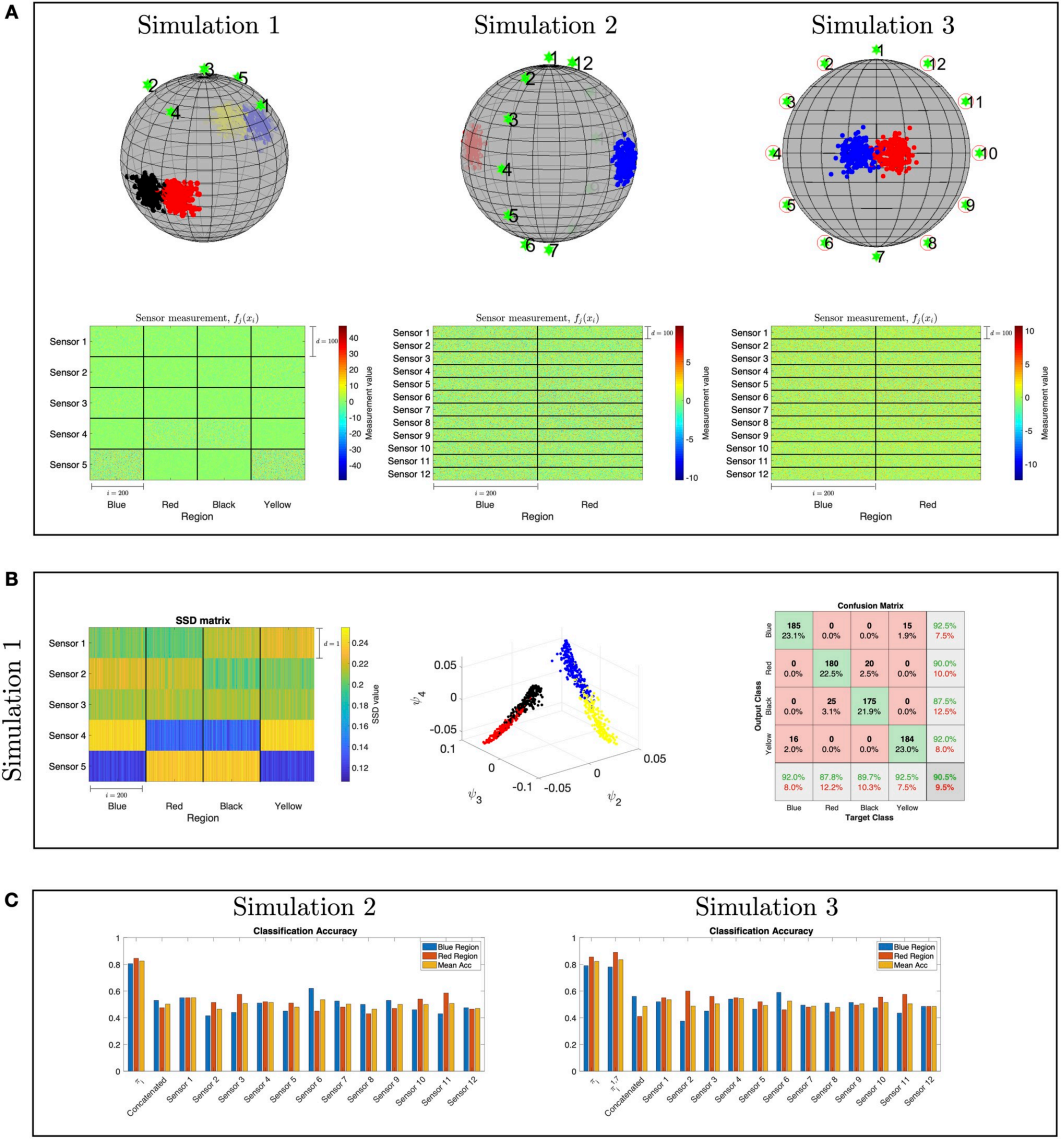
Illustration of localization toy problems. (A) The sensors and objects locations on a sphere are marked by green stars and dots, respectively. The multi-sensor observations correspond to Simulation 1 (left), Simulation 2 (middle), and Simulation 3 (right). (B) Results of the application of our approach to Simulation 1: the SSDs obtained by the proposed method (left), the diffusion maps embedding (middle), and the localization confusion matrix obtained by a 10-fold cross-validation with an RBF SVM classifier (right). (C) A comparison between the localization accuracy obtained by the proposed method based on SSDs and the localization accuracy obtained based on the output of each sensor as well as the concatenation of the output from all the sensors. The localization accuracy is the number of correctly identified positions divided by the true number of total positions in each region.

In Fig 4A, we present our setting consisting of objects and sensors located on and near a sphere, respectively. The objects positions are marked by dots, the different regions are marked by different colors (red, black, blue and yellow), and the sensors positions are marked by green stars. At the bottom, we present the (high-dimensional) sensor observations. Each block consists of 100 × 200 scalar observations corresponding to the observations of a single sensor from each region, where *d* = 100 is the dimension of each observation and 200 is the number of the positions per region. Visually, it is evident that distinguishing between the different regions merely based on these observations is non trivial.

For illustration purposes, we view the problem as a classification problem, where given the high-dimensional multi-feature observations, the task is to identify in which region the object at *x*_*i*_ resides.

The observations from each sensor consisting of 800 object positions from four regions were processed in two stages: first, a 3D embedding is constructed by applying diffusion maps to the high-dimensional observations, and second, an RBF SVM is applied to the obtained embedding in order to classify the region. To evaluate the classifiers, we perform a 10-fold cross-validation. A similar two-step procedure is applied to the concatenation of the observations from all the sensors.

Table 2 presents the resulting classification accuracy, i.e., the number of correctly classified positions divided by the total number of positions in each region. The presented results are obtained using a 10-fold cross-validation. We observe that none of the sensors enables an accurate classification. Moreover, we show in Table 2 that a naïve concatenation of the observations from all the sensors do not yield a good classification either.

**Table 2:** Localization accuracy from measurements of Simulation 1.

Region	Concatenated sensors	Sensor 1	Sensor 2	Sensor 3	Sensor 4	Sensor 5
Blue	43%	46%	18%	44.5%	58%	40.5%
Red	54%	15.5%	52.5%	45.5%	48.5%	55%
Black	46.5%	49.5%	29%	40%	37.5%	42%
Yellow	38.5%	33.5%	52%	30.5%	42.5%	50.5%

The performances obtained by the 10-fold cross-validations are presented. The percentages indicate the number of correctly classified positions divided by the true number of total positions in each class.

Seemingly, in order to mitigate the problem, we could simply represent the objects positions by a vector of the variance of the observations. However, it would require prior knowledge about the sensing model, whereas our approach is model-free.

In Fig 4B, we present the classification results obtained by the proposed method. In the diffusion maps embedding constructed from the SSDs, we observe a clear separation between the four regions. Finally, in the confusion matrix of the classification, we observe that the proposed method leads to significantly better classification results compared to the results in Table 2. This demonstrates the importance of taking into account the mutual-relationships between the sensor observations, rather than processing the nominal values of the observations directly, which in this case, give rise to correct identification of the four regions.

#### Simulation 2

The main purpose of this simulation is to demonstrate Proposition 2 from Binary hypothesis testing.

Consider *n* = 400 positions, ${\left\{x_{i}\right\}}_{i=1}^{400}$, such that $x_{i}\in S^{2}\subset R^{3}$, which are sampled from two different regions on the sphere. Suppose that the positions of the objects follow a bimodal distribution as depicted in the middle-top figure in Fig 4A: an object is located in the blue region with probability $\frac{1}{2}$ and in the red region with probability $\frac{1}{2}$. The two regions represent the two hypotheses, $H_{1}$ and $H_{2}$, where each region consists of 200 positions. Note the symmetry in this setting, that is, the distances from the blue and red regions to the sensors are approximately the same.

Here, we have 12 sensor observations ${\left\{f_{j}(x_{i})\right\}}_{j=1}^{12}$, measuring the positions of the objects, located at *s*_*j*_. The multi-feature observations are random samples from

$$\begin{array}{c} {\left\{f_{j}\left(x_{i}\right)\right\}}_{j=1}^{12}∼\frac{1}{2}N\left(0,\Sigma_{1}\right)+\frac{1}{2}N\left(0,\Sigma_{2}\right), \end{array}$$ (54)

where $f_{j}\left(x_{i}\right)\in R^{100}$, the standard deviation is $\sigma_{j}^{i}=20\exp(-∥x_{i}-s_{j}{∥}_{2}^{2})$, and the covariance $\Sigma_{k,l}∼\left|N\left(0,0.5\right)\right|$ for $\lfloor \frac{k}{100}\rfloor ,\lfloor \frac{l}{100}\rfloor \in \left[1,\ldots ,12\right]$.

In this simulation, a direct computation can show that Assumptions (A.1) and (A.2) hold. Specifically, we note that

$$\frac{\left|\right|{\left\langle g^{2}\right\rangle }_{\Omega_{r}}-{\left\langle g^{2}\right\rangle }_{\Omega_{b}}{\left|\right|}_{2}}{\sqrt{\left|\right|{\left\langle g^{2}\right\rangle }_{\Omega_{r}}{\left|\right|}_{2}\cdot \left|\right|{\left\langle g^{2}\right\rangle }_{\Omega_{b}}{\left|\right|}_{2}}}=0.03$$

and

$$\frac{{||\left\langle \Sigma \right\rangle }_{\Omega_{r}}-{\left\langle \Sigma \right\rangle }_{\Omega_{b}}{\left|\right|}_{F}}{\sqrt{{||\left\langle \Sigma \right\rangle }_{\Omega_{r}}{\left|\right|}_{F}\cdot {||\left\langle \Sigma \right\rangle }_{\Omega_{b}}{\left|\right|}_{F}}}=0.02,$$

where Ω_*r*_ and Ω_*b*_ denote the sets of red or blue object locations, respectively. In addition, the conditions of Special Case 1 are satisfied, and thus, the total variation of these sensor observations is zero. In other words, using a single sensor is insufficient to distinguish between the two regions. Conversely, we show that since the SSDs take into account the covariance information between the sensors, they allow us to make this distinction.

In Fig 4C, we present a comparison between the classification results obtained by our approach using SSDs and the classification results obtained by using the output of each sensor as well as the concatenation of the output from all the sensors. The results are evaluated with a 10-fold cross-validation. We observe that the classification obtained by proposed algorithm is significantly better than the classification obtained using the “raw” sensor outputs.

#### Simulation 3

This simulation demonstrates Proposition 3 from Binary hypothesis testing. Consider *n* = 400 positions, ${\left\{x_{i}\right\}}_{i=1}^{400}$, such that $x_{i}\in S^{2}\subset R^{3}$, which are sampled from two different regions on the sphere, as depicted in the right-top figure in Fig 4A. The rest of the setting remains as described in Simulation 2.

Note that here, only two sensor observations satisfy the conditions of Special Case 1, namely, $\sigma_{1}^{1}=\sigma_{1}^{2}$ and $\sigma_{7}^{1}=\sigma_{7}^{2}$, and thus, the total variation of only these two sensor observations is zeros.

For each data point, we compute the difference between the left-hand side and the right-hand side of the inequality in Proposition 2 per sensor. Then, in the right-top figure in Fig 4A, we circle the sensors attaining the largest difference. As expected, we observe that the circled sensors are positioned in non-symmetric orientations with respect to the locations of the two regions.

Similarly to the previous simulation, we compare the classification results obtained by our proposed method to the results attained by applying the classification directly to the observations from each sensor separately and to the concatenation of the observations from all the sensors. In addition, we also compute the results of Algorithm 1 applied to all the sensors except Sensor 1 and Sensor 7, which are the least contributing according to the inequality in Proposition 2.

The classification results presented in Fig 4C imply that by removing the least contributing sensors, namely Sensor 1 and Sensor 7, the recomputed SSDs, denoted by $\pi_{i}^{1,7}$, lead to slightly improved classification accuracy.

## Supporting information

S1 Appendix Detailed derivation of Proposition 1. (PDF)

S1 Fig Examples of expression levels of CD4, LAG3, B7H4 and CD20 before and after a median filter. (A): images from Dataset 1 with no filter (top) and after application of median filter (bottom). (B): same as (A) but for Dataset 2.
(TIFF)

S2 Fig Heatmaps showing the accuracy of the prediction of treatment response obtained by RBF SVM classifiers based on different choices of hyperparameters. The prediction is based on A: the proposed method in Box 1, B: DM, C: HKS and D: WKS. At each panel, the prediction results for Dataset 1 and Dataset 2 are presented.
(TIFF)

S3 Fig Treatment response prediction based on an ensemble of classifiers. The confusion matrices obtained by combining the RBF SVM classifiers based on different choices of hyperparameters. The combined parameter values are presented at the top. A: Dataset 1. B: Dataset 2.
(TIFF)

S4 Fig Heatmaps showing the accuracy of the prediction of treatment response obtained by Random Forest classifiers based on different choices of hyperparameters. Same as S2 Fig, but the results are obtained by random forest (RF) classifiers.
(TIFF)

S5 Fig Heatmaps of the IMC data and the corresponding SSD features. A: Dataset 1. B: Dataset 2. Each heatmap is divided into 2 vertical blocks representing the data collection from non-responders and responders. Each column in the heatmaps on the left consists of the multi-feature observations at one ROI. The column is composed of observations of *m* = 29 markers, where each marker observation is represented by a vector of size of *b* × *b*, which is a column stack representation of the corresponding image patch. Each column in the heatmaps on the right consists of the SSD features of size *m* = 29 at one ROI. The hyperparameters used for extracting the SSD features are presented on the left.
(TIFF)

S6 Fig Run time analysis. The run time (in seconds) of the proposed method in Box 1 and the three competing methods applied to three different choices of the number of ROIs (patches) *N* in Dataset 1 and Dataset 2. The run time is computed separately for the two stages of the algorithms. It is based on a Matlab implementation running on a single core 2.2GHz i7 CPU on a Macbook Pro from mid 2015 with 16GB 1600 MHz DDR3 RAM. A: the run time of Stage 1 for Dataset 1. B: the run time of Stage 2 for Dataset 1. C: the run time of Stage 1 for Dataset 2. D: the run time of Stage 2 for Dataset 2.
(TIFF)

S7 Fig Multi-SSD. We illustrate this multiscale property using a 3D shape from Princeton ModelNet40 database [42]. Suppose the points on the shape are the graph nodes, and compute the SSD of the graph with different values of *ϵ*, where the *ϵ* are chosen in logarithmic spacing between [10^−3^, 10^1.5^]. The color represents the values of SSD with different scales *ϵ* computed based on data points from a 3D shape of flowers in a vase. The red color represents high values of SSD, and, the blue color represents the low value of SSD. We observe that ***π***_*i*_ highlights junctions or hubs (in red), as in [43], both at local and global scales, depending on *ϵ*. We also observe that when *ϵ* is small, the SSD highlights the neck of each flower. When gradually increasing the value of *ϵ*, we observe that the SSD transitions toward the center of the shape, representing the global hub of the shape.
(EPS)

S1 Matlab Code Localization toy examples code. The folder consists of a text file (readme.txt) and three Matlab scripts demonstrating the three simulations in Localization toy problem.
(ZIP)
