# Supplementary material for: Graph of graphs analysis for multiplexed data with application to imaging mass cytometry
Source: PLoS Comput Biol. 2021 Mar 29;17(3):e1008741. doi: 10.1371/journal.pcbi.1008741 (PMC8032202; doi:10.1371/journal.pcbi.1008741)
Supplement: S1 Appendix — (PDF) [file pcbi.1008741.s001.pdf]

Given S is the set of the measurement. Based on the Taylor expansion, the kernel density estimation (KDE) for the sample x_i:

$$\begin{aligned}
\mathrm{KDE}_i(k) &\simeq \sum_{l=1}^{m} \exp\bigg(-\frac{\mathbb{E}(||V_k^i - V_l^i||^2)}{2\epsilon}\bigg) \\
&= \sum_{l=1}^{m} \exp\bigg(-\frac{||\mu_k^i - \mu_l^i||^2 + \sigma_k^i + \sigma_l^i + 2\sigma_{k,l}^i}{2\epsilon}\bigg) \\
&\approx \sum_{l=1}^{m} \bigg(1 - \frac{||\mu_k^i - \mu_l^i||^2 + \sigma_k^i + \sigma_l^i + 2\sigma_{k,l}^i}{2\epsilon}\bigg) \\
&= m - \frac{m}{2\epsilon}(\mu_k^i)^2 - \frac{1}{2\epsilon}\sum_{l=1}^{m}(\mu_l^i)^2 - \frac{m}{2\epsilon}\sigma_k^i - \frac{1}{2\epsilon}\sum_{l=1}^{m}\sigma_l^i + \frac{1}{2\epsilon}\sum_{l=1}^{m} 2\mu_k^i\mu_l^i - \frac{1}{\epsilon}\sum_{l=1, l\neq k}^{m}\sigma_{k,l}^i \\
&= m - \frac{1}{2\epsilon}\sum_{l=1}^{m}\bigg((\mu_l^i)^2 + \sigma_l^i\bigg) - \frac{m}{2\epsilon}\bigg((\mu_k^i)^2 + \sigma_k^i - \frac{2\mu_k^i}{m}\sum_{l=1}^{m}\mu_l^i\bigg) - \frac{1}{\epsilon}\sum_{l=1, l\neq k}^{m}\sigma_{k,l}^i \\
&= m - \frac{1}{2\epsilon}\sum_{l=1}^{m}\bigg((\mu_l^i)^2 + \sigma_l^i\bigg) - \frac{m}{2\epsilon}\bigg((\mu_k^i)^2 + \sigma_k^i - \frac{2\mu_k^i}{m}\sum_{l=1}^{m}\mu_l^i + (\frac{1}{m}\sum_{l=1}^{m}\mu_l^i)^2 - (\frac{1}{m}\sum_{l=1}^{m}\mu_l^i)^2\bigg) - \frac{1}{\epsilon}\sum_{l=1, l\neq k}^{m}\sigma_{k,l}^i \\
&= m - \frac{1}{2\epsilon}\sum_{l=1}^{m}\bigg((\mu_l^i)^2 + \sigma_l^i\bigg) + \frac{m}{2\epsilon}(\frac{1}{m}\sum_{l=1}^{m}\mu_l^i)^2 - \frac{m}{2\epsilon}\bigg((\mu_k^i - \frac{1}{m}\sum_{l=1}^{m}\mu_l^i)^2 + \sigma_k^i\bigg) - \frac{1}{\epsilon}\sum_{l=1, l\neq k}^{m}\sigma_{k,l}^i \\
&= m - \frac{m}{2\epsilon}\bigg(\frac{1}{m}\sum_{l=1}^{m}\bigg((\mu_l^i)^2 + \sigma_l^i\bigg)\bigg) + \frac{m}{2\epsilon}(\mu^i)^2 - \frac{m}{2\epsilon}\bigg((\mu_k^i - \mu^i)^2 + \sigma_k^i\bigg) - \frac{m}{\epsilon}\bigg(\frac{1}{m}\sum_{l=1, l\neq k}^{m}\sigma_{k,l}^i\bigg) \\
&= m - \frac{m}{2\epsilon}\bigg(\frac{1}{m}\sum_{l=1}^{m}\bigg((\mu_l^i)^2 + \sigma_l^i - (\mu^i)^2\bigg)\bigg) - \frac{m}{2\epsilon}\bigg(\frac{(\mu_k^i - \mu^i)^2}{(\mu_k^i)^2}(\mu_k^i)^2 + \sigma_k^i\bigg) - \frac{m}{\epsilon}\bigg(\frac{1}{m}\sum_{l=1, l\neq k}^{m}\sigma_{k,l}^i\bigg) \\
&= m - \frac{m}{2\epsilon}\bigg(\frac{1}{m}\sum_{l=1}^{m}\bigg(\boldsymbol{g}_i^2(l) + \sigma_l^i\bigg)\bigg) - \frac{m}{2\epsilon}\bigg(\boldsymbol{g}_i^2(k) + \sigma_k^i\bigg) - \frac{m}{\epsilon}\bigg(\frac{1}{m}\sum_{l=1, l\neq k}^{m}\sigma_{k,l}^i\bigg)
\end{aligned} \tag{1}$$

Note that $\boldsymbol{\pi}_i(k) \propto \boldsymbol{D}_i\mathbf{1}$, that is,

$$\boldsymbol{\pi}_i(k) = \frac{\mathrm{KDE}_i(k)}{\sum_{k=1}^{m}\mathrm{KDE}_i(k)}. \tag{2}$$

Therefore,

$$
\begin{aligned}
\boldsymbol{\pi}_i(k) &= \frac{m - \frac{m}{2\epsilon}\Bigg(\frac{1}{m}\sum_{l=1}^{m}\Bigg(\boldsymbol{g}_i^2(l) + \sigma_l^i\Bigg)\Bigg) - \frac{m}{2\epsilon}\Bigg(\boldsymbol{g}_i^2(k) + \sigma_k^i\Bigg) - \frac{m}{\epsilon}\Bigg(\frac{1}{m}\sum_{l=1,l\neq k}^{m}\sigma_{k,l}^i\Bigg)}{\sum_{k=1}^{m}\Bigg(m - \frac{m}{2\epsilon}\Bigg(\frac{1}{m}\sum_{l=1}^{m}\Bigg(\boldsymbol{g}_i^2(l) + \sigma_l^i\Bigg)\Bigg) - \frac{m}{2\epsilon}\Bigg(\boldsymbol{g}_i^2(k) + \sigma_k^i\Bigg) - \frac{m}{\epsilon}\Bigg(\frac{1}{m}\sum_{l=1,l\neq k}^{m}\sigma_{k,l}^i\Bigg)\Bigg)} \\
&= \frac{m - \frac{m}{2\epsilon}\Bigg(\frac{1}{m}\sum_{l=1}^{m}\Bigg(\boldsymbol{g}_i^2(l) + \sigma_l^i\Bigg)\Bigg) - \frac{m}{2\epsilon}\Bigg(\boldsymbol{g}_i^2(k) + \sigma_k^i\Bigg) - \frac{m}{\epsilon}\Bigg(\frac{1}{m}\sum_{l=1,l\neq k}^{m}\sigma_{k,l}^i\Bigg)}{= m^2 - \frac{m^2}{\epsilon}\Bigg(\frac{1}{m}\sum_{l=1}^{m}\Bigg(\boldsymbol{g}_i^2(l) + \sigma_l^i\Bigg)\Bigg) - \frac{m^2}{\epsilon}\Bigg(\frac{1}{m^2}\sum_{k=1}^{m}\sum_{l=1,l\neq k}^{m}\sigma_{k,l}^i\Bigg)} \\
&= \frac{\frac{m}{2} - \frac{m}{2\epsilon}\Bigg(\frac{1}{m}\sum_{l=1}^{m}(\boldsymbol{g}_i^2(l) + \sigma_l^i)\Bigg) - \frac{m}{2\epsilon}\Bigg(\frac{1}{m^2}\sum_{k=1}^{m}\sum_{l=1,l\neq k}^{m}\sigma_{k,l}^i\Bigg)}{m^2 - \frac{m^2}{\epsilon}\Bigg(\frac{1}{m}\sum_{l=1}^{m}(\boldsymbol{g}_i^2(l) + \sigma_l^i)\Bigg) - \frac{m^2}{\epsilon}\Bigg(\frac{1}{m^2}\sum_{k=1}^{m}\sum_{l=1,l\neq k}^{m}\sigma_{k,l}^i\Bigg)} \\
&\quad + \frac{\frac{m}{2} - \frac{m}{2\epsilon}\Bigg(\boldsymbol{g}_i^2(k) + \sigma_k^i\Bigg) - \frac{m}{\epsilon}\Bigg(\frac{1}{m}\sum_{l=1,l\neq k}^{m}\sigma_{k,l}^i\Bigg) + \frac{m}{2\epsilon}\Bigg(\frac{1}{m^2}\sum_{k=1}^{m}\sum_{l=1,l\neq k}^{m}\sigma_{k,l}^i\Bigg)}{m^2 - \frac{m^2}{\epsilon}\Bigg(\frac{1}{m}\sum_{l=1}^{m}(\boldsymbol{g}_i^2(l) + \sigma_l^i)\Bigg) - \frac{m^2}{\epsilon}\Bigg(\frac{1}{m^2}\sum_{i=1,j\neq i}^{m}\sum_{l=1}^{m}\sigma_{k,l}^i\Bigg)} \\
&= \frac{1}{2m} + \frac{\frac{m}{2} - \frac{m}{2\epsilon}\Bigg(\boldsymbol{g}_i^2(k) + \sigma_k^i\Bigg) - \frac{m}{\epsilon}\Bigg(\frac{1}{m}\sum_{l=1,l\neq k}^{m}\sigma_{k,l}^i\Bigg) + \frac{m}{2\epsilon}\Bigg(\frac{1}{m^2}\sum_{k=1}^{m}\sum_{l=1,l\neq k}^{m}\sigma_{k,l}^i\Bigg)}{m^2 - \frac{m^2}{\epsilon}\Bigg(\frac{1}{m}\sum_{l=1}^{m}(\boldsymbol{g}_i^2(l) + \sigma_l^i)\Bigg) - \frac{m^2}{\epsilon}\Bigg(\frac{1}{m^2}\sum_{k=1}^{m}\sum_{l=1,l\neq k}^{m}\sigma_{k,l}^i\Bigg)} \\
&= \frac{1}{2m} + \frac{\frac{1}{2} - \frac{1}{2\epsilon}\Bigg(\boldsymbol{g}_i^2(k) + \sigma_k^i\Bigg) - \frac{1}{\epsilon}\Bigg(\frac{1}{m}\sum_{l=1,l\neq k}^{m}\sigma_{k,l}^i\Bigg) + \frac{1}{2\epsilon}\Bigg(\frac{1}{m^2}\sum_{k=1}^{m}\sum_{l=1,l\neq k}^{m}\sigma_{k,l}^i\Bigg)}{m - \frac{m}{\epsilon}\Bigg(\frac{1}{m}\sum_{l=1}^{m}(\boldsymbol{g}_i^2(l) + \sigma_l^i)\Bigg) - \frac{m}{\epsilon}\Bigg(\frac{1}{m^2}\sum_{k=1}^{m}\sum_{l=1,l\neq k}^{m}\sigma_{k,l}^i\Bigg)} \\
&= \frac{1}{2m} + \frac{\epsilon - \Bigg(\boldsymbol{g}_i^2(k) + \sigma_k^i\Bigg) - 2\Bigg(\frac{1}{m}\sum_{l=1,l\neq k}^{m}\sigma_{k,l}^i\Bigg) + \Bigg(\frac{1}{m^2}\sum_{k=1}^{m}\sum_{l=1,l\neq k}^{m}\sigma_{k,l}^i\Bigg)}{2\epsilon m - 2m\Bigg(\frac{1}{m}\sum_{l=1}^{m}(\boldsymbol{g}_i^2(l) + \sigma_l^i)\Bigg) - 2m\Bigg(\frac{1}{m^2}\sum_{k=1}^{m}\sum_{l=1,l\neq k}^{m}\sigma_{k,l}^i\Bigg)} \\
&= \frac{1}{2m} + \frac{\epsilon - \Bigg(\boldsymbol{g}_i^2(k) + \sigma_k^i\Bigg) - 2\langle\boldsymbol{\Sigma}_{k,\cdot}^i\rangle_S + \langle\boldsymbol{\Sigma}^i\rangle_S}{2\epsilon m - 2m\Bigg(\langle\boldsymbol{g}_i^2\rangle_S + \langle\boldsymbol{\Sigma}^{\boldsymbol{i}}\rangle_S\Bigg)}
\end{aligned} \tag{3}
$$
