# Supplementary figures and images for: Graph of graphs analysis for multiplexed data with application to imaging mass cytometry
Source: PLoS Comput Biol. 2021 Mar 29;17(3):e1008741. doi: 10.1371/journal.pcbi.1008741 (PMC8032202; doi:10.1371/journal.pcbi.1008741)

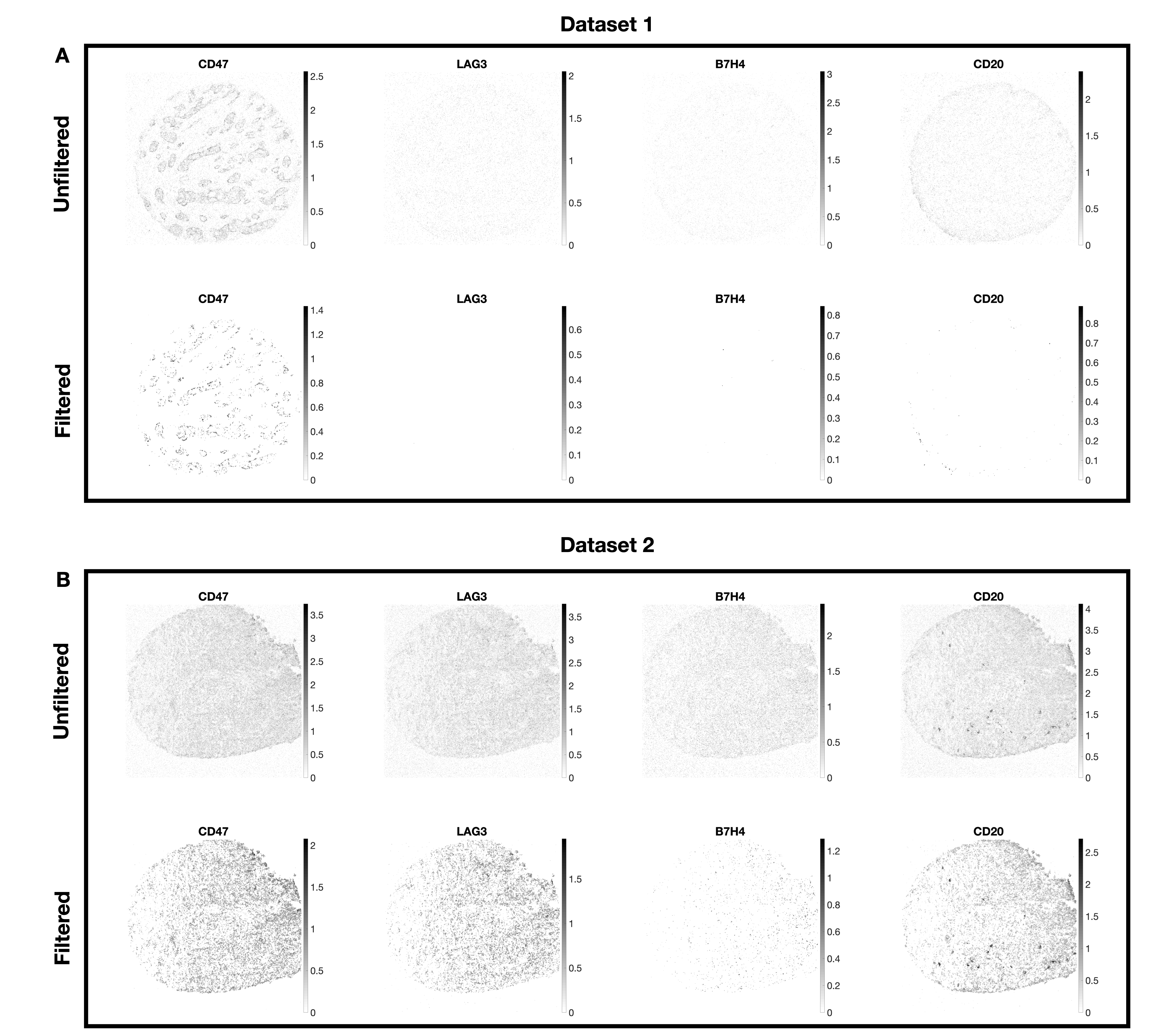

Supplement: S1 Fig — (A): images from Dataset 1 with no filter (top) and after application of median filter (bottom). (B): same as (A) but for Dataset 2. (TIFF) [file pcbi.1008741.s002.tiff]

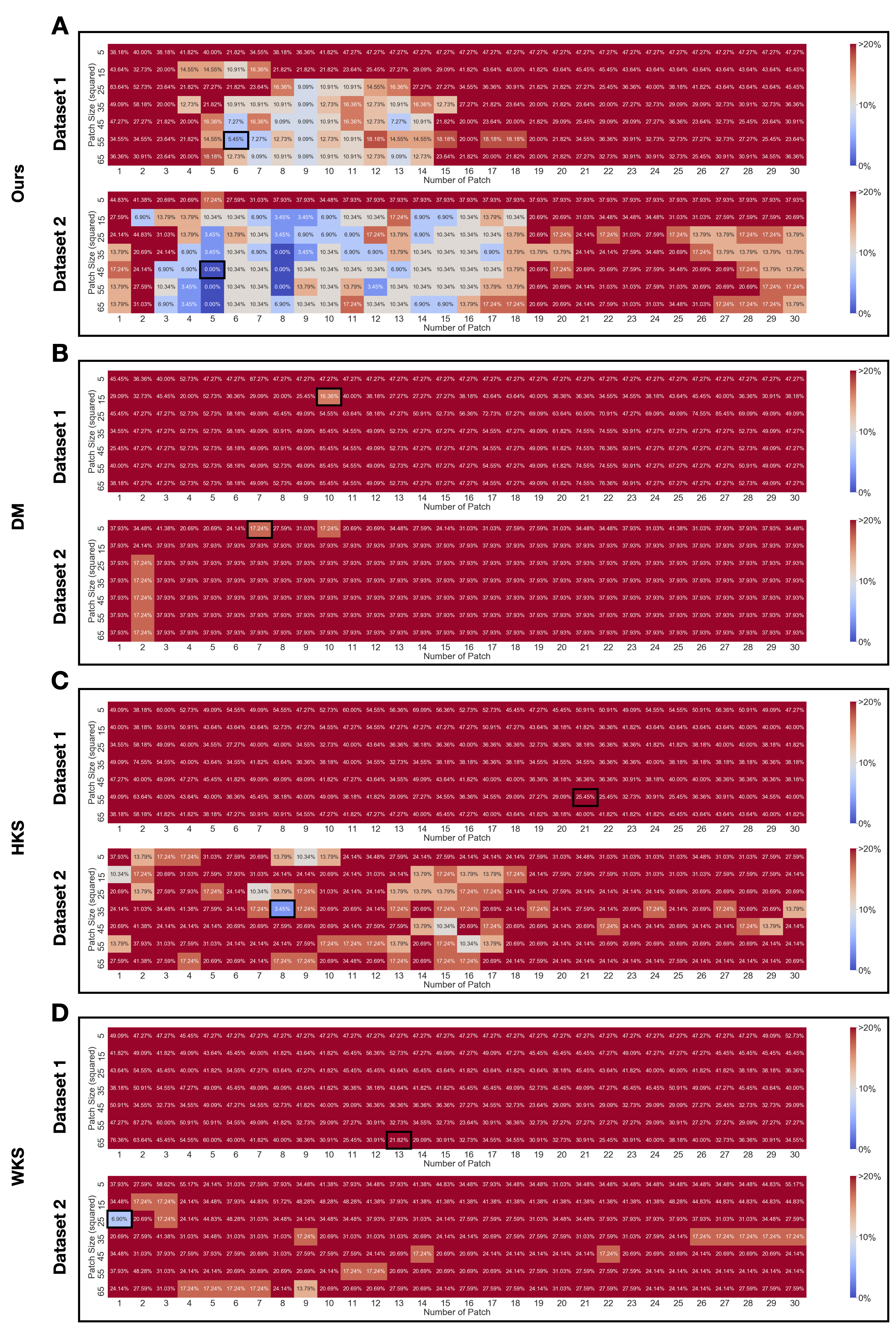

Supplement: S2 Fig — The prediction is based on A: the proposed method in Box 1, B: DM, C: HKS and D: WKS. At each panel, the prediction results for Dataset 1 and Dataset 2 are presented. (TIFF) [file pcbi.1008741.s003.tiff]

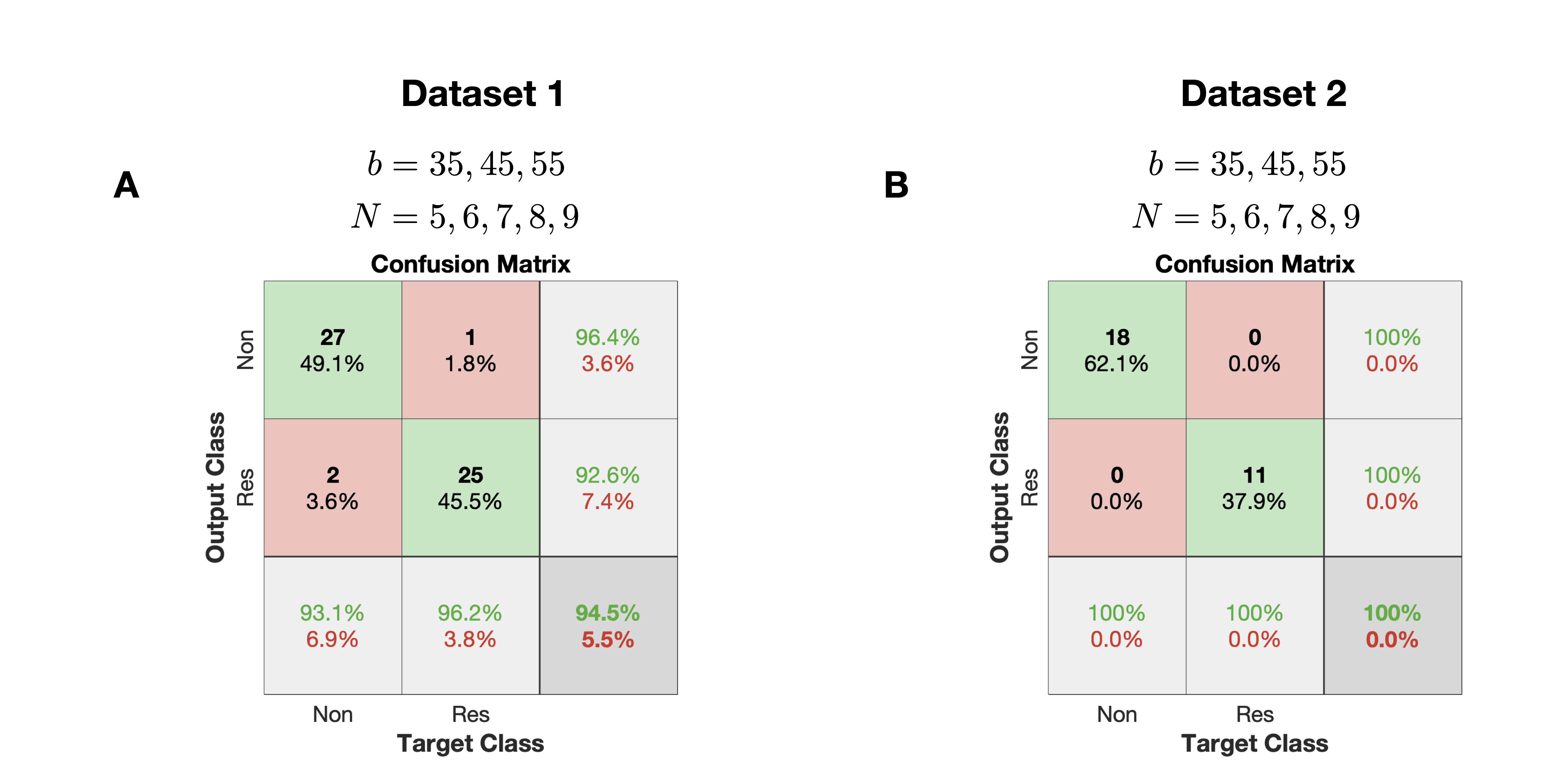

Supplement: S3 Fig — The confusion matrices obtained by combining the RBF SVM classifiers based on different choices of hyperparameters. The combined parameter values are presented at the top. A: Dataset 1. B: Dataset 2. (TIFF) [file pcbi.1008741.s004.tiff]

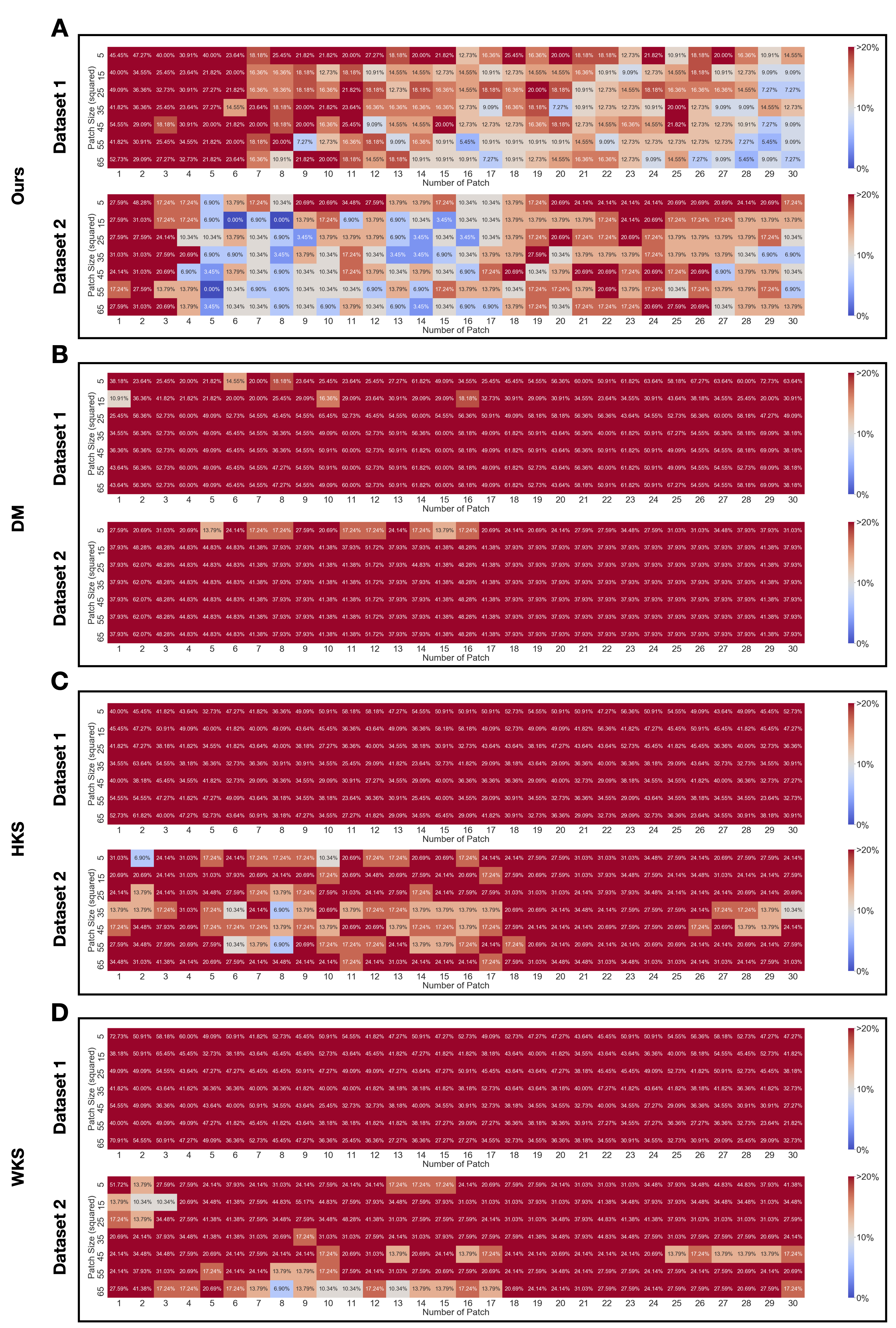

Supplement: S4 Fig — Same as S2 Fig, but the results are obtained by random forest (RF) classifiers. (TIFF) [file pcbi.1008741.s005.tiff]

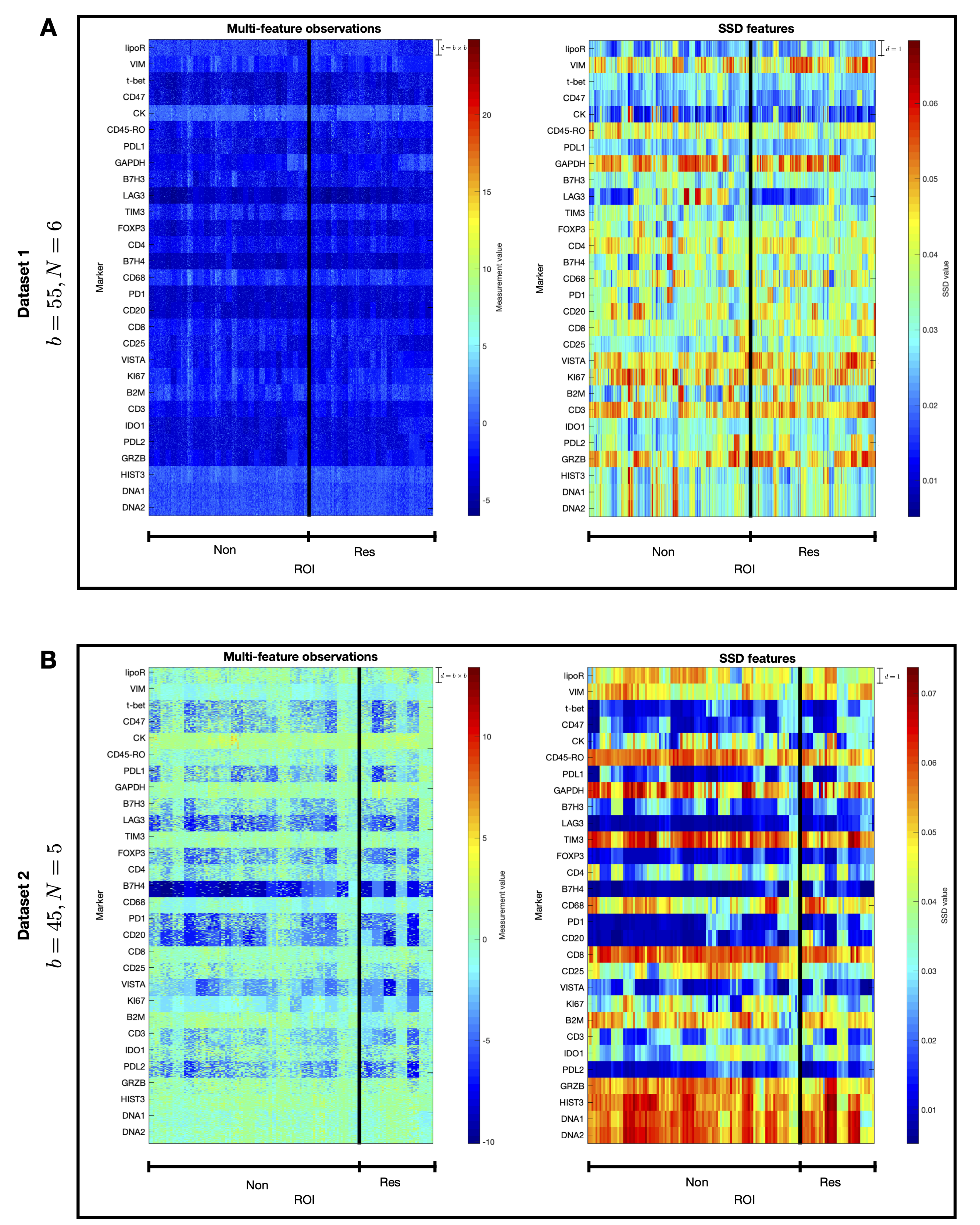

Supplement: S5 Fig — A: Dataset 1. B: Dataset 2. Each heatmap is divided into 2 vertical blocks representing the data collection from non-responders and responders. Each column in the heatmaps on the left consists of the multi-feature observations at one ROI. The column is composed of observations of m = 29 markers, where each marker observation is represented by a vector of size of b × b, which is a column stack representation of the corresponding image patch. Each column in the heatmaps on the right consists of the SSD features of size m = 29 at one ROI. The hyperparameters used for extracting the SSD features are presented on the left. (TIFF) [file pcbi.1008741.s006.tiff]

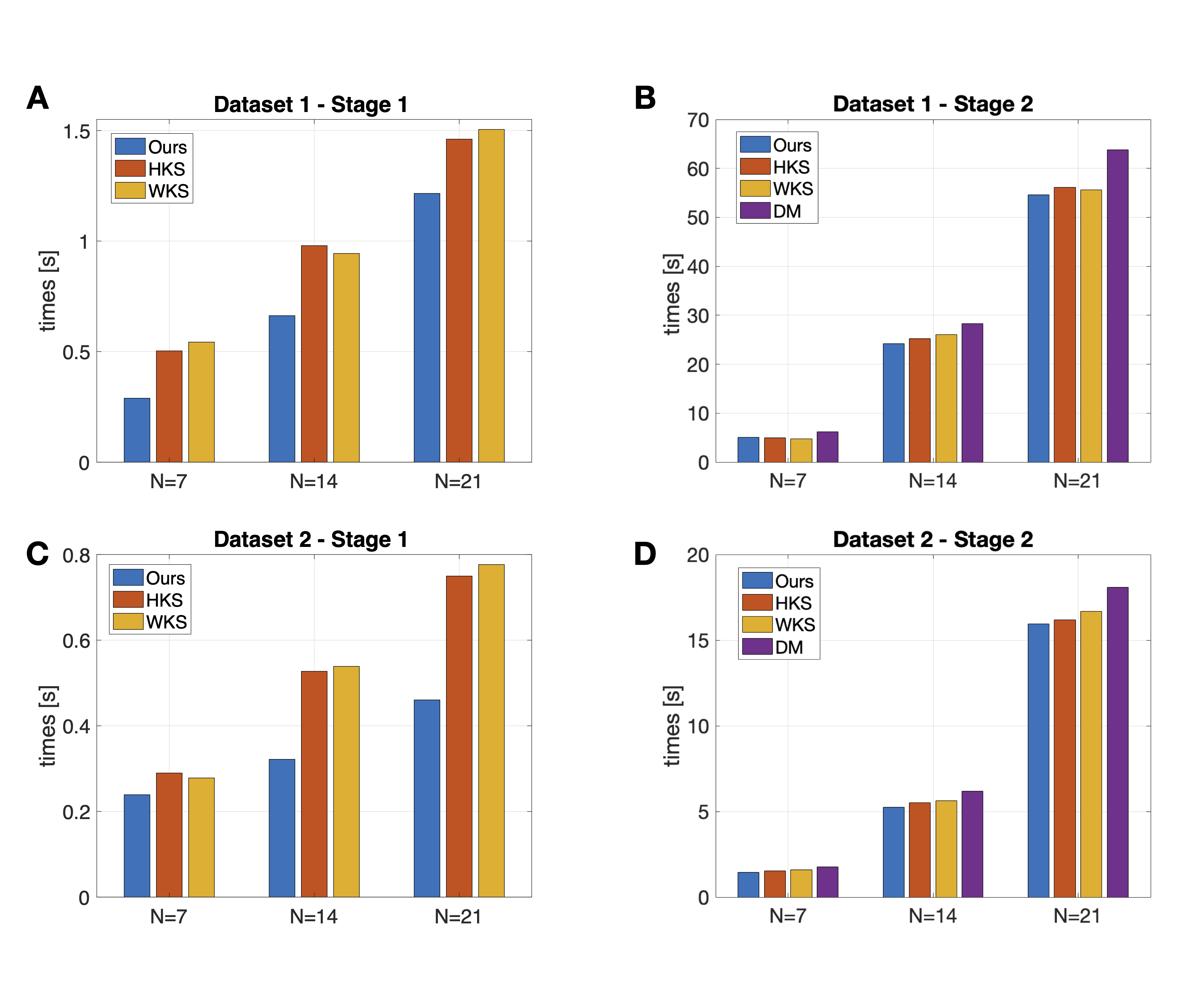

Supplement: S6 Fig — The run time (in seconds) of the proposed method in Box 1 and the three competing methods applied to three different choices of the number of ROIs (patches) N in Dataset 1 and Dataset 2. The run time is computed separately for the two stages of the algorithms. It is based on a Matlab implementation running on a single core 2.2GHz i7 CPU on a Macbook Pro from mid 2015 with 16GB 1600 MHz DDR3 RAM. A: the run time of Stage 1 for Dataset 1. B: the run time of Stage 2 for Dataset 1. C: the run time of Stage 1 for Dataset 2. D: the run time of Stage 2 for Dataset 2. (TIFF) [file pcbi.1008741.s007.tiff]
